# Pharmacogenomics of in vitro response of the NCI-60 cancer cell line panel to Indian natural products

**DOI:** 10.1186/s12885-022-09580-7

**Published:** 2022-05-07

**Authors:** Hari Sankaran, Simarjeet Negi, Lisa M. McShane, Yingdong Zhao, Julia Krushkal

**Affiliations:** Biometric Research Program, Division of Cancer Treatment and Diagnosis, National Cancer Institute, National Institutes of Health, 9609 Medical Center Drive, Rockville, MD 20850 USA

**Keywords:** Ayurveda, Natural products, Drug response, Cancer cell lines, NCI-60, Gene expression, Single nucleotide variation

## Abstract

**Background:**

Indian natural products have been anecdotally used for cancer treatment but with limited efficacy. To better understand their mechanism, we examined the publicly available data for the activity of Indian natural products in the NCI-60 cell line panel.

**Methods:**

We examined associations of molecular genomic features in the well-characterized NCI-60 cancer cell line panel with in vitro response to treatment with 75 compounds derived from Indian plant-based natural products. We analyzed expression measures for annotated transcripts, lncRNAs, and miRNAs, and protein-changing single nucleotide variants in cancer-related genes. We also examined the similarities between cancer cell line response to Indian natural products and response to reference anti-tumor compounds recorded in a U.S. National Cancer Institute (NCI) Developmental Therapeutics Program database.

**Results:**

Hierarchical clustering based on cell line response measures identified clustering of *Phyllanthus* and cucurbitacin products with known anti-tumor agents with anti-mitotic mechanisms of action. Curcumin and curcuminoids mostly clustered together. We found associations of response to Indian natural products with expression of multiple genes, notably including SLC7A11 involved in solute transport and ATAD3A and ATAD3B encoding mitochondrial ATPase proteins, as well as significant associations with functional single nucleotide variants, including BRAF V600E.

**Conclusion:**

These findings suggest potential mechanisms of action and novel associations of in vitro response with gene expression and some cancer-related mutations that increase our understanding of these Indian natural products.

**Supplementary Information:**

The online version contains supplementary material available at 10.1186/s12885-022-09580-7.

## Background

### History of Ayurveda

Ayurveda is a traditional system of medicine that originated around 3000–4000 BCE, which utilizes Indian natural products (INP) derived mainly from plants to treat “imbalances” in the body aiming to cure a variety of diseases, including cancer [1]. In the Ayurvedic system of herbal medicine, there are 3 main physiologic states called doshas which are based on several phenotypic (body frame, weight, facial features) and mental (memory, emotional lability) factors. A fundamental belief in Ayurvedic medicine is that an imbalance in these doshas leads to disease and illness, which are purported to be corrected by a combination of these herbal remedies [2].

Historical references in Ayurvedic text contain some of the first descriptions of cancer (blood and soft tissue) and their successful treatment with a combination of INPs administered via oral and topical routes [2]. However, results reported in these historical references are difficult to replicate due to the use of multiple herbal products in combination, a difference in basic disease terminology, and heterogeneity in preparation of the herbal compounds [3, 4]. Despite the uncertain efficacy of these INPs, Ayurvedic medications have been reported to be used by as many as 20–40% of patients with cancer in India as they are believed to prevent chemotherapy-related toxicity, boost immunity, and slow tumor growth [5, 6]. Knowledge of the putative anticancer mechanisms of action of individual molecular compounds comprising the INPs is incomplete, however some in vitro and in vivo data for several commonly used INPs exist and are discussed below.

### Examples of Indian natural products

Curcumin is a bioactive polyphenol that is the most common curcuminoid, a group of compounds that impart a yellow color to *Curcuma longa* (turmeric). Curcumin has generated a lot of interest as an INP with possible chemo-preventative, anticancer, and anti-inflammatory properties, highlighting the difficulty of defining a specific indication due to its description as a panacea [7]. Some reports have demonstrated the modest activity of curcumin to induce apoptosis in cancer cell lines, its role in enhancing response to cisplatin, and its anti-inflammatory properties [7, 8]. These findings have led to many trials including active clinical trials in the US (NCT02064673, NCT02944578, NCT02782949) exploring the role of curcumin as a chemo-preventative agent in preventing gastric cancer, cervical intraepithelial neoplasia, and the recurrence of prostate cancer.

Neem (*Azadirachta indica)* is another commonly used herbal product that has several component INPs with reported anticancer properties, which highlights the difficulty in isolating active INP compounds. Nimbolide is a terpenoid lactone derived from Neem that induces apoptosis in pancreatic cancer cells through reactive oxygen species (ROS) generation and upregulation of pro-apoptotic proteins [9]. Gedunine, a pentacyclic triterpenoid derived from Neem, has also demonstrated activity in pancreatic cancer through inhibition of the sonic hedgehog pathway [10]. These mechanisms of action of multiple INPs from the same herbal product make it difficult to attribute the activity of INPs, which is further complicated as many patients taking INPs receive combinations of several herbal products.

Amla (*Phyllanthus emblica)*, a.k.a. Indian gooseberry, is part of the genus *Phyllanthus*, which has been used in traditional herbal medicine to treat multiple ailments. The *Phyllanthus* genus includes several species (e.g., *P. niruri*, *P. urinaria*, *P. fraternus*, etc.) which have been used to treat a wide range of ailments from diabetes to renal calculi [11]. Although anecdotal reports of use of Amla to treat cancer are lacking, some active molecules in Amla have been studied more extensively, including quercetin. Quercetin, a polyphenolic flavonoid derived from *P. emblica,* has been shown to attenuate tumor growth in breast and pancreatic cancer models through multiple mechanisms including growth signal inhibition of the PI3K pathway and tyrosine kinase inhibition [12].

Cucurbitacins are a group of compounds characterized by a tripterpene hydrocarbon. which are found in over 40 species, including Indian plants such as Brahmi (*Bacopa monnieri*) and bitter gourd (*Momordica charantia*) [13]. These plants, which are known for their bitter taste due to the cucurbitacins, are purported to prevent cancer and are administered orally as a liquid formulation. While cucurbitacin B is one of the more extensively studied cucurbitacins, its putative anticancer mechanism of action is not well defined; however this product is thought to be involved in JAK/STAT pathway inhibition and F-actin cytoskeleton disruption [14].

While putative anti-cancer mechanisms of action have been suggested for commonly used INPs as detailed above, these data are often limited to in vitro response in one or a few cell lines*.* Data regarding rarer INPs including plumbagin (*Plumbago zeylanica*), alizarin (*Rubia cordifolia*), and Achilleol A (*Achillea odorata*) are limited or have not yet been reported [15]. Analysis of data from a large database of cell line assay results such as the NCI-60 cancer cell line panel data, for the purpose of determining a mechanism of action, may improve our understanding of these INPs.

### NCI-60 cell line panel

Our overall strategy to explore the possible mechanisms of action of INPs was to compare patterns of cell line response to each INP with publicly available data to those for standard reference anticancer compounds and to identify clusters (subtrees) of INPs with similar patterns of response across the NCI-60 cell lines. Next, we examined the association of gene expression levels and of clinically or biologically important single nucleotide variants (SNVs) with response to individual INPs. We also examined how the molecular features associated with tumor cell line responses to individual INPs were distributed among the INP subtrees that had similar patterns of response. Lastly, we investigated the biological pathways representing the gene expression patterns that were associated with different INP subtrees. These analyses provided new insights into potential mechanisms of actions of the INPs.

To examine the activity of INPs in tumor cells, we analyzed publicly available data from the NCI-60 cancer cell line panel. The NCI-60 initiative was started by the U.S. National Cancer Institute (NCI) in 1989 with the purpose of screening candidate anti-cancer compounds on 60 cancer cell lines representing 10 different tumor types. Over 100,000 compounds have been screened to date, including INPs and well-characterized reference compounds approved for clinical use (e.g., paclitaxel, methotrexate, and other agents) [16–18]. The Developmental Therapeutics Program (DTP) of the NCI screens these compounds using a single high-dose test to meet pre-specified minimum inhibition criteria and subsequently screens each compound in a 5-dose screen using a 48 h endpoint measured by a Sulforhodamine B stain [18]. Data recorded by the screen include GI50, IC50, LC50, and total growth inhibition (TGI) cell response data which are used to generate unique patterns across cell lines [17–19]. To interrogate this rich dataset, the COMPARE algorithm was developed to allow comparisons of response patterns (across cell lines) of synthetic and natural products of interest with standard reference compounds to help determine their putative mechanisms of actions [16].

Additionally**,** molecular features of the NCI-60 cell lines have been extensively characterized. Their gene expression, whole exome sequencing, and other molecular data have been made publicly available [20, 21]. These data were integrated into online databases and made available through CellMiner and CellMinerCDB data portals, which allow access to gene expression, genetic variation, and drug sensitivity data [22, 23]. Measures of response of the cell lines to a large number of drugs and investigational compounds, including some natural products, are also publicly available from the NCI DTP NCI-60 Growth Inhibition data repository. Combined, these data provide an opportunity to assess gene-drug relationships. Thus, the NCI-60 resource offers a robust dataset that may be interrogated to increase our understanding of INPs and their mechanisms of action.

## Methods

**Fig. 1:**
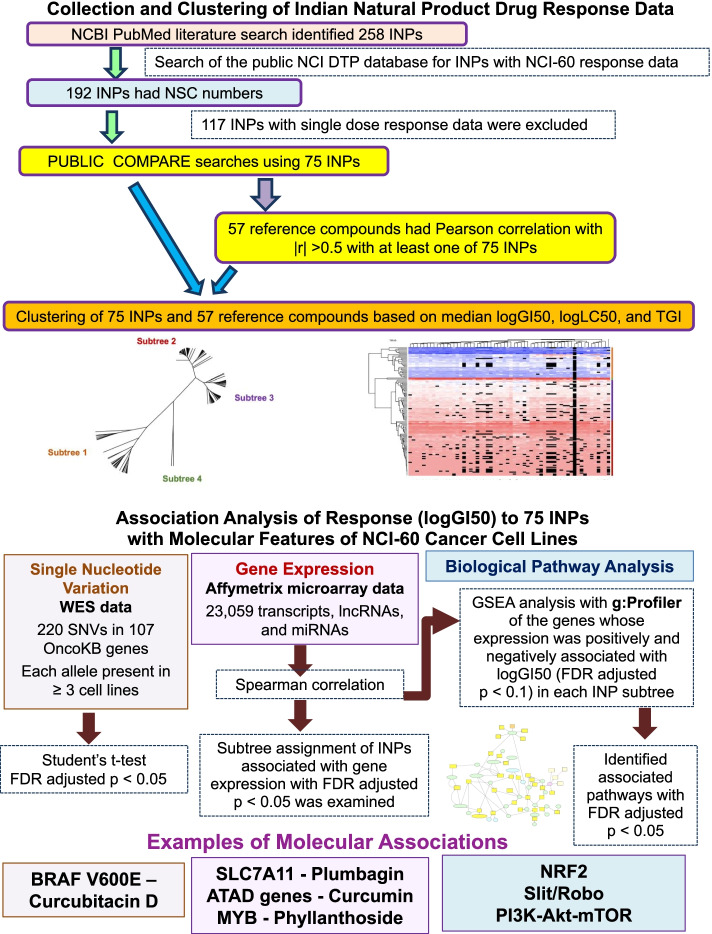
The workflow of the steps in the analysis of the response of NCI-60 cancer cell lines to the Indian Natural Products and of the association of that response with molecular features of the NCI-60 cell lines. Details of each analysis are provided in the Methods section. *INP* Indian Natural Products, *GSEA* Gene set enrichment analysis, *NCBI* National Center for Biotechnology Information, *TGI* Total Growth Inhibition

Figure 1 summarizes the workflow of the steps of the analyses in this study.

### Collection of Indian natural products and reference compounds with cell line response data

A biomedical literature search in PubMed at the National Center for Biotechnology Information (NCBI) using keywords “Ayurveda” AND “cancer” AND “review” was conducted to identify Ayurvedic herbs of interest, with a total of 170 publications found. Each publication was manually reviewed. Among them, 25 publications contained a comprehensive description of one or more Ayurvedic herbs and their specific INPs that are commonly used by Ayurvedic practitioners in cancer treatment. These INPs were included in subsequent searches. All INPs identified in our manual curation were then searched in PubMed for evidence of any activity in cancer cell lines and were compiled, resulting in the total of 258 INPs.

The NCI DTP screening program uses a special identifier, called an NSC number, for each compound screened in the NCI-60 cell line panel. Those INPs obtained from our literature search that did not have NSC numbers (*n* = 66) were excluded from further analysis. The unique NSC numbers for the remaining INPs (*n* = 192) identified from biomedical literature were interrogated using the NCI PUBLIC COMPARE portal for available GI50 data (https://dtp.cancer.gov/public_compare) [24, 25]. Each GI50 value represents sensitivity of an NCI-60 cell line to a particular compound, calculated as the concentration producing 50% growth inhibition that is derived from the 5-concentration screen of each compound at 48 h after incubation [18]. Those INPs with only single dose response data (*n* = 117) were excluded. The remaining INPs (*n* = 75) were used as input for separate queries in the NCI PUBLIC COMPARE portal. The public version of the NCI PUBLIC COMPARE database does not store the taxonomy and global locations of the original source products for the database compounds. The queries use Pearson correlation analysis to compare the vector of GI50 values across the NCI-60 cell line panel for each input INP to the vector of GI50 values for available COMPARE reference antitumor agents (including approved agents, e.g., methotrexate and vincristine, and experimental agents). We used a cutoff of the absolute value for a pairwise Pearson correlation coefficient |r|> 0.5 to select the reference compounds with similar GI50 response profiles to each input INP.

The NSC numbers of the 75 INPs and the 57 reference compounds that were correlated with at least one of those 75 INPs with |r|> 0.5 (Table 1) were used to download publicly available -log_10_ GI50 data (negative log_10_ GI50, referred as NLOGGI50 in the downloadable dataset) from the static public release at the DTP website NCI-60 Growth Inhibition data repository (https://wiki.nci.nih.gov/display/NCIDTPdata/NCI-60+Growth+Inhibition+Data). This dataset is currently available under previous releases (filename: NCI60_GI50_2016b.zip, June 2016 release downloaded on March 4, 2020). Details of the sample handling, preparation and cell line testing methods followed to generate the data in this repository are described elsewhere [19]. The NLOGGI50 values were multiplied by -1 in order to convert them to log_10_GI50, a measure of cell line response to treatment. Here and below, we refer to these measures as logGI50. All logGI50 values that were not available were set to missing. The term “compound” is used to describe the INPs and reference compounds with available logGI50 data. As multiple experiments had been run for each compound, the median logGI50 was calculated, using replicate experiments, for each cell line-compound pair. These median logGI50 values for each NCI-60 cell line were computed for all 132 compounds using 15,199 experiment records. The majority of the data were screened in molar units, except for the product of *Ricinus communis* (NSC 15384), which had the units in μg/ml and was not included in the clustering analysis for that reason. A more detailed description of the public COMPARE algorithm and the NCI-60 cell line panel can be found elsewhere [16].

**Table 1:** Indian Natural Products and reference compounds with the absolute value of the pairwise Pearson correlation coefficient |r| between their logGI50 values > 0.5

NSC	Compound Name	Type (Ayurveda/Reference)	Plant Name/Reference Product Mechanism	logGI50 Dendrogram Subtree	Same subtree based on logLC50 vs logGI50	Same subtree based on TGI vs logGI50
15384	*Ricinus communis*	Ayurveda	*Ricinus communis* (Castor)	Not included in clustering	Not included in clustering	Not included in clustering
740	Methotrexate	Reference	antimetabolite	Subtree 1	No	No
58514	Chromomycin A3	Reference	antitumor antibiotics	Subtree 1	Yes	Yes
332596	Rhizoxin	Reference	antitumor antibiotics	Subtree 1	No	Yes
332598	Rhizoxin	Reference	antitumor antibiotics	Subtree 1	Yes	Yes
143925	Pekilocerin A	Ayurveda	*Calotropis* (Madar)	Subtree 1	Yes	Yes
144153	Datiscoside	Ayurveda	*Cordia dichotoma* (Indian Cherry)	Subtree 1	No	No
49451	Curcubitacin B	Ayurveda	*Cucurbitae* family	Subtree 1	No	Yes
94743	Cucurbitacin A	Ayurveda	*Cucurbitae* family	Subtree 1	Yes	No
106399	Cucurbitacin E	Ayurveda	*Cucurbitae* family	Subtree 1	Yes	Yes
112167	Elatericin B	Ayurveda	*Cucurbitae* family	Subtree 1	Yes	Yes
308606	Cucurbitacin D	Ayurveda	*Cucurbitae* family	Subtree 1	No	No
521777	Elatericin B	Ayurveda	*Cucurbitae* family	Subtree 1	No	No
352122	Trimetrexate	Reference	antimetabolite	Subtree 1	No	No
139105	Soluble Baker's Antifol	Reference	antimetabolite	Subtree 1	No	No
123127	Doxorubicin (Adriamycin)	Reference	antitumor antibiotic	Subtree 1	No	No
337766	Bisantrene hydrochloride	Reference	antitumor antibiotic	Subtree 1	Yes	Yes
49842	Vinblastine sulfate	Reference	mitotic inhibitor	Subtree 1	Yes	Yes
67574	Vincristine sulfate	Reference	mitotic inhibitor	Subtree 1	No	No
90636	Vinleurosine Sulfate	Reference	mitotic inhibitor	Subtree 1	No	No
125973	Paclitaxel (Taxol)	Reference	mitotic inhibitor	Subtree 1	No	No
153858	Maytansine	Reference	mitotic inhibitor	Subtree 1	No	Yes
141537	Anguidine	Reference	not defined	Subtree 1	Yes	Yes
165563	Bruceantin	Reference	antitumor antibiotic	Subtree 1	Yes	Yes
325319	Didemnin B	Reference	Protein synthesis inhibitor	Subtree 1	Yes	Yes
328426	Phyllanthoside	Ayurveda	*Phyllanthus* genus	Subtree 1	Yes	Yes
342443	S3'-desacetyl-Phyllanthoside	Ayurveda	*Phyllanthus* genus	Subtree 1	No	Yes
3053	Actinomycin D	Reference	antitumor antibiotic	Subtree 1	Yes	Yes
19912	Cryptopleurine	Ayurveda	*Tylophora* Alkaloids	Subtree 1	Yes	Yes
76387	Tylophorin	Ayurveda	*Tylophora Indica*	Subtree 1	No	Yes
717335	Tylophorin	Ayurveda	*Tylophora Indica*	Subtree 1	No	Yes
375575	Cyclopentenylcytosine	Reference	antimetabolite	Subtree 1	No	No
156236	Achillin	Ayurveda	*Achillea odorata *(Yarrow)	Subtree 2	Yes	Yes
710351	Achilleol A	Ayurveda	*Achillea odorata* (Yarrow)	Subtree 2	Yes	Yes
26428	Esculetin	Ayurveda	*Aesculus Hippocastanum* (Horse Chestnut)	Subtree 2	Yes	Yes
750	Busulfan	Reference	alkylator	Subtree 2	Yes	Yes
344007	Piperazine alkylator	Reference	alkylator	Subtree 2	Yes	Yes
353451	Mitozolamide	Reference	alkylator	Subtree 2	Yes	Yes
409962	BCNU	Reference	alkylator	Subtree 2	Yes	Yes
227189	Aloin	Ayurveda	*Aloe* (Kumariasava)	Subtree 2	Yes	Yes
5605	Benzalacetone	Ayurveda	*Alpinia Galanga* (Asian Ginger)	Subtree 2	Yes	Yes
139490	Emofolin sodium	Reference	antimetabolite	Subtree 2	No	No
224131	PALA	Reference	antimetabolite	Subtree 2	No	No
731917	Calendulaglycoside B	Ayurveda	*Calendula* *officinalis* (pot marigold)	Subtree 2	Yes	Yes
62794	Beta carotene	Ayurveda	*Daucas carota* (Carrot)	Subtree 2	Yes	Yes
2819	Cianidol	Ayurveda	Catechin (*Bergenia ciliate*)	Subtree 2	Yes	Yes
643032	M-Phenoxy-alpha-phenylcinnamonitrile	Ayurveda	*Cinnamonum* (Cinnamon)	Subtree 2	Yes	Yes
643033	P-Acetoxy-alpha-diethylphosphono-cinnamonitrile	Ayurveda	*Cinnamonum* (Cinnamon)	Subtree 2	Yes	Yes
643160	3-Bromo-4-dimethylamino-alpha-benzoyl cinnamonitrite	Ayurveda	*Cinnamonum* (Cinnamon)	Subtree 2	Yes	Yes
643167	3,4-Methylenedioxy-alpha-benzoyl cinnamonitrile	Ayurveda	*Cinnamonum* (Cinnamon)	Subtree 2	Yes	Yes
643181	3,4,5-Trimethoxy-alpha-benzoyl cinnamonitrile	Ayurveda	*Cinnamonum* (Cinnamon)	Subtree 2	Yes	Yes
643183	3-Methoxy-4-hydroxy-alpha-benzoylcinnamonitrile	Ayurveda	*Cinnamonum *(Cinnamon)	Subtree 2	Yes	Yes
643185	3,5-Dimethoxy-alpha-phenylcinnamonitrile	Ayurveda	*Cinnamonum* (Cinnamon)	Subtree 2	Yes	Yes
643190	3-Methoxy-4-benzyloxy-alpha-benzoylcinnamonitrile	Ayurveda	*Cinnamonum* (Cinnamon)	Subtree 2	Yes	Yes
643764	O-Methoxy-alpha-benzoylcinnamonitrile	Ayurveda	*Cinnamonum* (Cinnamon)	Subtree 2	Yes	Yes
643772	O-Fluoro-alpha-benzoyl cinnamonitrile	Ayurveda	*Cinnamonum* (Cinnamon)	Subtree 2	Yes	Yes
184734	Cucurbitacin I	Ayurveda	*Cucurbitae* family	Subtree 2	Yes	Yes
682343	Curcumenol	Ayurveda	*Curcuma zedoaria* (White turmeric)	Subtree 2	Yes	Yes
327430	Resveratrol	Ayurveda	Darakchasava (*Vitis* *vinifera*)	Subtree 2	Yes	Yes
285115	DQ1	Ayurveda	Datura	Subtree 2	Yes	Yes
90487	Lupeol	Ayurveda	*Hemidesmus indicus* (Indian Sarsaparilla)	Subtree 2	Yes	Yes
57197	Caffeic Acid	Ayurveda	Honey, coffee	Subtree 2	Yes	Yes
32065	Hydroxyurea	Reference	ribonucleotide reductase inhibitor	Subtree 2	No	No
51143	IMPY	Reference	ribonucleotide reductase inhibitor	Subtree 2	No	No
253272	Caracemide	Reference	ribonucleotide reductase inhibitor	Subtree 2	Yes	Yes
291643	Pyrimidine-5-glycodialdehyde	Reference	ribonucleotide reductase inhibitor	Subtree 2	Yes	Yes
118994	Diglycoaldehyde	Reference	antimetabolite	Subtree 2	No	No
126849	3-deazauridine	Reference	antimetabolite	Subtree 2	No	No
218321	2'-deoxycoformycin	Reference	antimetabolite	Subtree 2	Yes	Yes
37364	O6-methylguanine	Reference	antimetabolite	Subtree 2	Yes	Yes
322921	Pibenzimol hydrochloride	Reference	topoisomerase inihibitor	Subtree 2	Yes	Yes
73754	Fluorodopan	Reference	alkylator	Subtree 2	Yes	Yes
303861	L-cysteine analogue	Reference	inhibitory amino acid analog	Subtree 2	Yes	Yes
844	Nesol	Ayurveda	Limonene (Citrus)	Subtree 2	Yes	Yes
368675	Azadirachtin	Ayurveda	*Azadiractha indica* (Neem)	Subtree 2	Yes	Yes
150014	Hydrazine sulfate	Reference	not defined	Subtree 2	No	No
293015	Flavone acetic acid ester	Reference	not defined	Subtree 2	Yes	Yes
343513	Dihydrolenperone	Reference	not defined	Subtree 2	Yes	Yes
407300	Crocetin	Ayurveda	Saffron	Subtree 2	Yes	Yes
178886	Paeony root	Ayurveda	*Paeonia officinalis* (Peony)	Subtree 2	Yes	Yes
619043	Phyllanthin	Ayurveda	*Phyllanthus* genus	Subtree 2	Yes	Yes
619044	Hypophyllanthin	Ayurveda	*Phyllanthus *genus	Subtree 2	Yes	Yes
9219	Quertine	Ayurveda	*Phyllanthus* genus	Subtree 2	Yes	Yes
7212	Alizarin	Ayurveda	*Rubia cordifolia* (Red madder)	Subtree 2	Yes	Yes
8096	Harzol	Ayurveda	*Saraca asoca* (Ashoka)	Subtree 2	Yes	Yes
284356	Mitindomide	Reference	topoisomerase inihibitor	Subtree 2	Yes	Yes
22842	Cumostrol	Ayurveda	*Trifolium pratens*e (Red clover)	Subtree 2	Yes	Yes
407290	Myricitin	Reference	not defined	Subtree 2	Yes	Yes
79037	CCNU	Reference	alkylator	Subtree 3	No	No
95441	Methyl-CCNU	Reference	alkylator	Subtree 3	No	No
167780	Asaley	Reference	alkylator	Subtree 3	No	Yes
330500	Macbecin II	Reference	antitumor antibiotics	Subtree 3	Yes	Yes
113497	Gedunine	Ayurveda	*Azadirachta indica *(Neem)	Subtree 3	No	No
309909	Nimbolide	Ayurveda	*Azadirachta indica *(Neem)	Subtree 3	Yes	Yes
87868	Phenethyl mustard oil	Ayurveda	*Brasicaceae* and *Fabaceae* (Watercress)	Subtree 3	Yes	Yes
708791	Bulbophyllanthrone	Ayurveda	*Bulbophyllum odaratissimum *(Orchid)	Subtree 3	Yes	Yes
652892	Butein	Ayurveda	*Butea monosperma* (Palash)	Subtree 3	No	No
731920	Calendulaglycoside B-6'-O-butyl ester	Ayurveda	*Calendula officinalis* (Pot marigold)	Subtree 3	No	No
731921	Calendulaglycoside D2	Ayurveda	*Calendula officinalis* (Pot marigold)	Subtree 3	Yes	Yes
731922	Calendulaglycoside D-6'-O-methyl ester	Ayurveda	*Calendula officinalis* (Pot marigold)	Subtree 3	Yes	Yes
26727	Cycvalon	Ayurveda	*Curcuma* genus (Turmeric)	Subtree 3	Yes	Yes
643023	Alpha-Phenyl-2,5-dimethoxy-alpha-cinnamonitrile	Ayurveda	*Cinnamonum* (Cinnamon)	Subtree 3	No	No
643769	O-Bromo-alpha-benzoyl cinnamonitrile	Ayurveda	*Cinnamonum* (Cinnamon)	Subtree 3	No	No
112166	Cucurbitacin K	Ayurveda	*Cucurbitae* family	Subtree 3	No	Yes
742019	Ethoxycurcumin tribenzimidazolmethylcarbonte	Ayurveda	*Curcuma* genus (Turmeric)	Subtree 3	Yes	Yes
742020	Ethoxycurcumin trithiadiazolaminomethylcarbonte	Ayurveda	*Curcuma* genus (Turmeric)	Subtree 3	Yes	Yes
742021	Curcumin tri adamantylaminoethylcarbonate	Ayurveda	*Curcuma* genus (Turmeric)	Subtree 3	No	No
742022	Curcumin tri trithiadiazolaminoethylcarbonate	Ayurveda	*Curcuma* genus (Turmeric)	Subtree 3	Yes	Yes
752571	Curcumin-difluorinated (CDF)	Ayurveda	*Curcuma* genus (Turmeric)	Subtree 3	Yes	Yes
705537	Daturaolone	Ayurveda	*Datura metel *(Datura)	Subtree 3	No	No
119875	Cisplatin	Reference	alkylator	Subtree 3	No	Yes
271674	Carboxyphthalatoplatinum	Reference	alkylator	Subtree 3	No	No
102816	5-azacytidine	Reference	DNA methyltransferase inhibitor	Subtree 3	No	Yes
91874	Emberine	Ayurveda	*Embelia Ribes* (False black pepper)	Subtree 3	No	Yes
180973	Tamoxifen	Reference	Estrogen receptor binder	Subtree 3	Yes	Yes
365798	Piceatannol	Ayurveda	*Vitis vinifera* (Grapes)	Subtree 3	No	No
674038	Gallocatechin	Ayurveda	*Punica granatum* (Pomegranate)	Subtree 3	No	No
383468	Andrographolide	Ayurveda	*Andrographis Paniculata* (Green chiretta)	Subtree 3	No	No
303812	Aphidicolin glycinate	Reference	DNA polymerase inhibitor	Subtree 3	No	No
133100	Rifamycin SV	Reference	inhibit DNA-dependent RNA polymerase	Subtree 3	No	No
83265	S-trityl-L-cysteine	Reference	mitotic inhibitor	Subtree 3	No	No
236613	Plumbagin	Ayurveda	*Plumbago* *zeylanica* (Chitrak)	Subtree 3	Yes	Yes
104801	Cytembena	Reference	antimetabolite	Subtree 3	No	No
163501	AT-125 (acivicin)	Reference	antimetabolite	Subtree 3	No	No
19893	5-fluorouracil	Reference	antimetabolite	Subtree 3	No	No
126771	Dichloroallyl lawsone	Reference	antimetabolite	Subtree 3	No	No
368390	DUP785 (brequinar)	Reference	antimetabolite	Subtree 3	No	No
77037	D-tetrandrine	Reference	not defined	Subtree 3	Yes	Yes
7616	Aconitic acid	Ayurveda	*Saccharum officinarum *(sugarcane)	Subtree 3	No	No
32982	Curcumin	Ayurveda	*Curcuma* genus (Turmeric)	Subtree 3	No	No
237020	Largomycin	Reference	not defined	Subtree 4	Yes	Yes
326231	L-Buthionine sulfoximine	Reference	not defined	Subtree 4	No	No

The column labeled Plant Name/Reference Product Mechanism shows the main mechanism of action of reference products and taxonomy for Ayurvedic compounds. More than one plant may contain the compound of interest

Cmpd: compound

logGI50 Dendrogram Subtree: shows subtree assignment of an INP or reference compound based on the clustering of logGI50 values

Same subtree based on logLC50 vs logGI50: indicates whether an INP or a reference compound showed a similar clustering with other INPs and compounds based on logLC50 values and was assigned to the subtree with the same number as compared to the subtree assignment based on logGI50 values

Same subtree based on TGI vs logGI50: indicates whether an INP or a reference compound showed a similar clustering with other INPs and compounds based on the total growth inhibition (TGI) values and was assigned to the subtree with the same number as compared to the subtree assignment based on logGI50 values

The product of the *Ricinus communis* (NSC 15384) was not included in the clustering analysis as its concentration units were different from those for other INPs

**Fig. 2:**
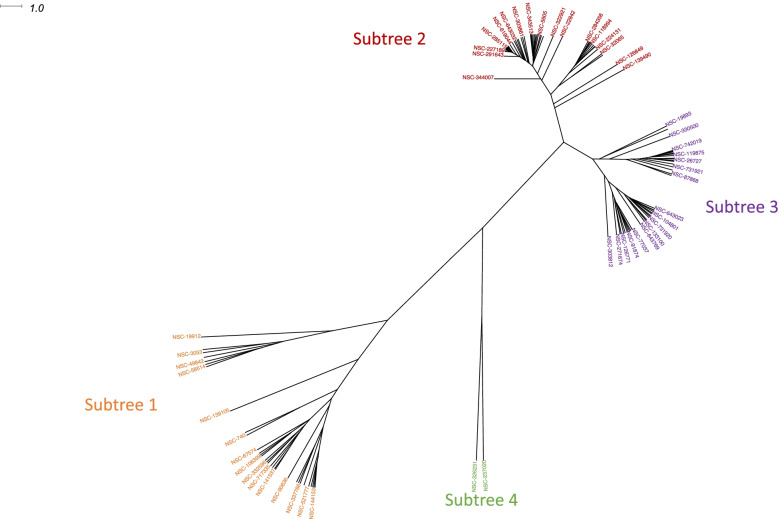
Hierarchical clustering of INPs and reference compounds based on their median logGI50 values across NCI60 cell lines. The tree was inferred using the UPGMA (‘average’) method and was based on Euclidean distances. The tree is presented as an unrooted radial phylogram. The scale in the top left corner is provided for the branch length, which were derived from Euclidean distances. Clustered products are displayed with sparse labeling, in which only a random subset of INP labels is displayed. Detailed information about the INPs in each subtree is provided in Table 1 and Supplementary Table 4

Clustering based on logGI50 is presented graphically in Fig. 2 and Supplementary Fig. 1. Clustering based on logLC50 and TGI is presented in Supplementary Figs. 2 and 3, respectively. Detailed comparison of differences among clustering based on different response measures is provided in Supplementary Table 5

### Hierarchical clustering of the logGI50, logLC50 and TGI values of INPs and reference compounds

In order to identify groups of INPs with similar patterns of activity in the NCI-60 cell line panel, we employed hierarchical clustering of the INPs. The initial clustering to identify groups of compounds with similar response patterns was based on the logGI50 values (Fig. 2). Reference compounds were also included in the clustering to provide information about possible mechanisms of action of each hierarchical cluster, or subtree, containing INPs with similar response. Clustering was based on pairwise Euclidean distances between each compound pair, which were calculated using the logGI50 values of the INPs and reference compounds in all 60 NCI-60 cell lines. A hierarchical tree based on these Euclidean distances was generated using the hclust package using the ‘average’, or UPGMA, option and exported for further visualization using the ape package [26]. Additionally, a 2-dimensional heatmap of the compounds and cell lines was generated from logGI50 values using heatmap.2 in the gplots package. We used RStudio v1.2.5033 for clustering analysis. Further visualization and graphical representation of the hierarchical clustering of all compounds and of their individual subtrees was done using Dendroscope version 3.7.2 [27].

To augment the analysis of clusters of INPs and reference compounds using logGI50 values, we also performed separate clustering of compounds using logLC50 and TGI values representing the 50% lethal concentration needed for the 50% cell kill and the concentration (also on the log_10_ scale) for the total inhibition of growth, respectively[18, 19, 25]. Both logLC50 and TGI values were downloaded from the December 2021 release of the NCI-60 Growth Inhibition Data (https://wiki.nci.nih.gov/NCIDTPdata/NCI-60+Growth+Inhibition+Data). Values for all INPs and reference compounds were extracted, and median values were computed as detailed above. Pairwise Euclidean distances were calculated, and unrooted radial hierarchical trees were generated using the methodology described above. These trees were visualized and compared to the tree inferred using logGI50 values (Fig. 2; Table 1). Subsequent analyses of association of INP response with gene expression, gene enrichment, and single nucleotide variation data were performed using logGI50 values as the primary endpoint measure.

### Analysis of association of gene expression with INP activity

To examine how NCI-60 cell line response to INPs may be influenced by molecular genetic features, we analyzed the association of median logGI50 values with NCI-60 molecular data. Pre-treatment gene expression data for the NCI-60 cell lines was downloaded from the CellMinerCDB resource [23, 28]. A more detailed description of the collection of molecular measures can be found in our previous publication [29]. For expression analysis, we used log_2_ transformed expression measures of 23,059 annotated transcripts, lncRNAs, and miRNAs which had been previously combined from five Affymetrix expression microarray platforms and normalized by the CellMiner development team [22]. Cell lines for which there were no drug response data (MDA-MB-468) or no gene expression data (MDA-N) were excluded (*n* = 2). For each gene-INP pair, Spearman correlation was computed to evaluate the association between pre-treatment gene expression and logGI50 in 58 cell lines. Benjamini–Hochberg procedure was applied to control the false discovery rate (FDR) across the 23,059 gene × 75 INP pairs. Gene-INP pairs with FDR-adjusted *p* < 0.05 were considered significant. A positive value of the Spearman correlation coefficient ρ indicated an association of higher gene expression with higher logGI50 values of an INP, i.e., with increased resistance to that INP. Similarly, negative values of ρ showed an association of higher gene expression with lower logGI50 values, i.e., with increased sensitivity to that INP. Here and below, the terms sensitivity and resistance were used to define the direction of the associations, as the analyses of logGI50 values were performed on the continuous scale. All genes with significant Spearman correlations were investigated to determine whether the gene involved in the gene-INP pair was associated with a known molecular mechanism of action of reference compounds that clustered in the same subtree with that INP.

### Gene set enrichment analysis

Gene set enrichment analysis was performed using g:Profiler (https://biit.cs.ut.ee/gprofiler/gost), which is a regularly updated web-based utility that includes annotated pathway gene sets from KEGG, Reactome, and WikiPathways [30]. Genes that were significantly associated with response to INPs (FDR adjusted *p* < 0.1) in each cluster were stratified to negatively and positively correlated groups (Supplementary Tables 1–3). GSEA analysis was performed on each gene group separately for each cluster, using the gene symbols as input for g:Profiler. A significance level for enriched pathways was set at *p* < 0.05 (FDR adjusted).

### Analysis of association of INP activity with single nucleotide variants

To examine the association between NCI-60 cell line response to INPs and specific DNA alterations of cancer genes that may affect cytotoxicity response, whole exome sequencing (WES) data were downloaded from the CellMiner data download portal [22, 31]. One cell line (MDA-N) which did not have drug response data was excluded, leaving a total of 59 cell lines available for analysis.

The data were filtered using a list of candidate genes and functionally relevant SNVs from OncoKB v. 1.17, a curated precision oncology knowledge base [32]. As outlined in our earlier report [29], the list consisted of variants classified by OncoKB at levels 1–4 of potential therapeutic action, R1 and R2 levels of resistance, and variants classified as “oncogenic” and “likely oncogenic”. After applying this filter to the CellMiner WES data, 1,586 protein changing SNVs in 280 genes across 59 NCI-60 cell lines were identified. These SNVs, which included nonsynonymous changes, frameshift variants, and variants involving the stop codon or the loss of a translational initiation codon start site, were additionally filtered to include only variants present in at least 3 NCI-60 cell lines, resulting in 107 genes with 220 SNVs across 59 cell lines. A Student’s *t*-test was used to compare logGI50 values between groups of NCI-60 cell lines defined by variant status, for each SNV-INP pair. A positive value of the *t*-statistic indicated an association of higher gene expression with higher logGI50 values of an INP, i.e. increased resistance to that INP, whereas a negative value of the *t*-statistic showed an association of higher gene expression with lower logGI50 values, i.e. increased sensitivity to that INP. All analyses of associations between response to the natural products and sequence variants were performed using the RStudio v1.0.153. Biological interpretation of significant SNV-response associations was based on SNV annotation in OncoKB, using its updated annotation of levels of functional and oncogenic SNV effects as of 03/25/2021, and on published reports in biomedical literature.

### Visualization of associations of response to INPs with molecular features and with cellular pathways in the NCI-60 cell lines

Visualization of significant associations (FDR adjusted *p* < 0.05) of logGI50 with gene expression and with single nucleotide variants, and of association of significantly upregulated and downregulated cellular pathways with INP subtrees was performed using Cytoscape v. 3.9.1 [33] and Microsoft Excel.

## Results

### Hierarchical clustering of Indian natural products and reference compounds based on the logGI50 measures

Figure 2 shows the hierarchical clustering of the Indian natural products and reference compounds based on their median logGI50 values, presenting the results as an unrooted radial phylogram. Clustering revealed 4 distinct subtrees. As Subtree 4 consisted of only reference products (NSC 326231 - L-buthionine sulfoximine, and NSC 237020 - largomycin), it was excluded from subsequent analysis. Supplementary Fig. 1 provides a heatmap showing the two-dimensional clustering of the NCI-60 cell lines and the INPs and reference compounds, clustered according to the similar patterns of cell line response to these compounds using logGI50 values. The similarities of logGI50 response patterns within each subtree may suggest similar potency of the INPs with their grouped reference products and possibly similar mechanisms of actions.

#### Subtree 1 (13 INPs and 18 reference products)

The reference compounds in this subtree have mainly anti-mitotic activity (vincristine sulfate, vinleurosine sulfate, vinblastine sulfate, paclitaxel); however, they also included some agents that act as DNA intercalators (doxorubicin) and anti-metabolites (methotrexate). Some INPs of the cucurbitacin family and its derivatives (Cucurbitacin A, B, D, E, L, datiscoside) affect mitotic spindles and delay mitoses leading to a G2/M phase cell cycle arrest of cancer cells [13, 14]. Phyllanthoside has been demonstrated to function both in vivo and in vitro as an inhibitor of eukaryotic protein synthesis by interfering with translation elongation, similar to the reference compound actinomycin D[34]. While a mechanism of action has not been clearly defined for tylophorin and its analog cryptoleurine, some experimental evidence points toward G1 arrest through cyclin A2 downregulation and VEGF2-mediated angiogenesis, which is not a known mechanism of any of the reference compounds correlated with its cytotoxicity [35, 36].

#### Subtree 2 (34 INPs and 22 reference products)

The 22 reference compounds in this subtree had many different mechanisms of action; however, the majority fit into either alkylators (piperazine, mitrozolamide, BCNU, busulfan), ribonucleoide reductase inhibitors (pyrimidine-5-glycodialdehyde, caracemide, IMPY, hydroxyurea), and broad inhibitors of RNA synthesis (diglycoaldehyde, 3-deazauridine). The 34 INPs included in this cluster consisted of a large group of cinnamon-based INPs and some *Phyllanthus* INPs.

#### Subtree 3 (25 INPs and 17 reference products)

The 17 reference compounds in this subtree consisted of a variety of alkylators (CCNU, methyl-CCNU, asaley), anti-metabolites (AT-125, 5-FU, DUP785, dichloroallyl lawsone), and DNA-crosslinking agents (carboxy-platinum). The 25 INPs included in Subtree 3 consisted of curcumin, curcuminoids, neem, and *Calendula* products.

### Hierarchical clustering of Indian natural products and reference compounds based on the logLC50 and TGI measures

Supplementary Figs. 2 and 3 show the hierarchical clustering of INPs and reference compounds based on their median logLC50 or TGI values, respectively. The trees inferred using logLC50 and TGI were similar to each other, except for 12 compounds. Both logLC50 and TGI trees were comprised of 5 distinct subtrees, as compared to 4 distinct subtrees in the logGI50 tree (Fig. 2, Supplementary Figs. 2–3). Table 1 provides information, for each INP and reference compound, whether a compound had a similar clustering with other compounds and was assigned to a subtree with the same number based on logLC50 and TGI as compared to the subtrees based on clustering of logGI50. Detailed comparison of the cluster assignment of the compounds based on different response measures is provided in Supplementary Table 5. Clustering which was based on TGI was more similar to logGI50-based clustering, whereas with the logLC50-based clustering more compounds showed differences from their logGI50-based cluster assignment (Supplementary Table 5). These patterns of similarity and difference between the three trees derived from different response measures may be explained by the fact that logGI50 and TGI both represent different degrees of growth inhibition, both being derived from the growth curve, whereas logLC50 is a different parameter representing a concentration needed to achieve 50% of cell kill [19]. Overall, the clustering was consistent for many INPs among the three difference response measures (Table 1 and Supplementary Table 5). It was less consistent for a number of reference compounds, possibly due to the higher potency of established anticancer drugs, which may result in their lower concentration needed to achieve total growth inhibition (TGI) or 50% lethal concentration (LC) as compared to the INPs. Seven reference compounds from subtree 2 of the logGI50 tree formed a separate cluster (subtree 5) in both TGI- and logLC50-based trees. Anti-mitotic reference compounds (e.g. vinblastine, vincristine) clustered closely together in logGI50 subtree 1, however they were not tightly clustered in both logLC50 and TGI trees. The cluster assignment of many INPs (e.g. cinnamon and turmeric) in both logLC50 and TGI trees was similar to that in the logGI50 tree.

### Association of cell line response to INPs with gene expression

Using pre-treatment gene expression data of 23,059 transcripts and the median logGI50 values of the 75 INPs, we conducted a Spearman correlation analysis that identified 204 natural product-gene pairs (including 190 unique genes and 28 unique INPs) that were statistically significant after adjusting for multiple testing (FDR adjusted *p* value < 0.05). All significant results are listed in Table 2 and summarized in a graphical format in Supplementary Fig. 4. Below we discuss some of the highly significant correlations of biologically important protein-coding genes.

**Table 2:** Significant associations of gene expression and logGI50 of Indian Natural Products in the NCI-60 cell line panel

NSC	Gene	Spearman ρ	Original *p* value	FDR adjusted *p* value	Dendrogram subtree	Active molecule
328426	MYB	-0.66	1.7e-08	0.004	Subtree 1	Phyllanthoside
328426	BEND7	0.65	2.8e-08	0.005	Subtree 1	Phyllanthoside
308606	ZBTB33	-0.67	1.0e-07	0.012	Subtree 1	Cucurbitacin D
328426	NHS	0.62	2.0e-07	0.014	Subtree 1	Phyllanthoside
328426	WWC1	0.61	3.0e-07	0.019	Subtree 1	Phyllanthoside
94743	TRMT112	0.65	4.0e-07	0.022	Subtree 1	Cucurbitacin A
328426	RBMS2	0.60	5.0e-07	0.022	Subtree 1	Phyllanthoside
342443	EHD2	0.66	6.0e-07	0.025	Subtree 1	S3’-desacetyl-phyllanthoside
328426	PROSER2	0.60	8.0e-07	0.027	Subtree 1	Phyllanthoside
328426	SMAP2	-0.60	8.0e-07	0.027	Subtree 1	Phyllanthoside
328426	BAGE	-0.59	8.0e-07	0.027	Subtree 1	Phyllanthoside
328426	C6orf89	-0.59	9.0e-07	0.027	Subtree 1	Phyllanthoside
328426	EGFR	0.59	1.0e-06	0.027	Subtree 1	Phyllanthoside
328426	PDLIM1	0.59	1.0e-06	0.027	Subtree 1	Phyllanthoside
94743	ZNF48	0.63	1.0e-06	0.027	Subtree 1	Cucurbitacin A
328426	CNPPD1	-0.59	1.1e-06	0.029	Subtree 1	Phyllanthoside
342443	CLMP	0.65	1.1e-06	0.029	Subtree 1	S3’-desacetyl-phyllanthoside
143925	ATP1A1	0.59	1.2e-06	0.030	Subtree 1	Pekilocerin A
342443	ADAM9	0.64	1.4e-06	0.031	Subtree 1	S3’-desacetyl-phyllanthoside
328426	MON1A	-0.58	1.4e-06	0.031	Subtree 1	Phyllanthoside
328426	ZNF319	0.58	1.6e-06	0.033	Subtree 1	Phyllanthoside
328426	HKDC1	0.58	1.8e-06	0.036	Subtree 1	Phyllanthoside
342443	CD151	0.64	2.0e-06	0.037	Subtree 1	S3’-desacetyl-phyllanthoside
328426	AJUBA	0.58	2.1e-06	0.038	Subtree 1	Phyllanthoside
112167	TLN1	-0.62	2.3e-06	0.040	Subtree 1	Elatericin B
328426	HESX1	-0.57	2.4e-06	0.040	Subtree 1	Phyllanthoside
328426	MMP24	0.57	2.5e-06	0.042	Subtree 1	Phyllanthoside
328426	TJP1	0.57	2.7e-06	0.043	Subtree 1	Phyllanthoside
328426	TNFRSF12A	0.57	2.7e-06	0.043	Subtree 1	Phyllanthoside
342443	ZFP36L1	0.63	2.9e-06	0.043	Subtree 1	S3’-desacetyl-phyllanthoside
342443	NNMT	0.63	3.0e-06	0.043	Subtree 1	S3’-desacetyl-phyllanthoside
328426	BIN1	0.57	3.0e-06	0.043	Subtree 1	Phyllanthoside
342443	MTCL1	0.63	3.1e-06	0.043	Subtree 1	S3’-desacetyl-phyllanthoside
342443	TNFRSF1A	0.62	3.8e-06	0.046	Subtree 1	S3’-desacetyl-phyllanthoside
342443	LOC101241902	0.62	3.8e-06	0.046	Subtree 1	S3’-desacetyl-phyllanthoside
328426	GOLGA6L5P	0.56	3.9e-06	0.046	Subtree 1	Phyllanthoside
521777	SLAMF6	0.56	3.9e-06	0.046	Subtree 1	Elatericin B
342443	UCKL1	-0.62	3.9e-06	0.046	Subtree 1	S3’-desacetyl-phyllanthoside
143925	ILF2P1	-0.56	4.0e-06	0.046	Subtree 1	Pekilocerin A
328426	NUAK2	0.56	4.0e-06	0.046	Subtree 1	Phyllanthoside
328426	PUS3	-0.56	4.1e-06	0.046	Subtree 1	Phyllanthoside
328426	C2CD2L	-0.56	4.3e-06	0.046	Subtree 1	Phyllanthoside
342443	NRP1	0.62	4.3e-06	0.046	Subtree 1	S3’-desacetyl-phyllanthoside
328426	LRRN4	0.56	4.3e-06	0.046	Subtree 1	Phyllanthoside
328426	SLC35F3	0.56	4.4e-06	0.046	Subtree 1	Phyllanthoside
328426	ZNF639	-0.56	4.6e-06	0.046	Subtree 1	Phyllanthoside
143925	FNIP1	0.56	4.7e-06	0.046	Subtree 1	Pekilocerin A
94743	ZNF629	0.60	4.8e-06	0.046	Subtree 1	Cucurbitacin A
328426	FRAT2	-0.56	5.6e-06	0.048	Subtree 1	Phyllanthoside
342443	NR2F2	0.61	5.8e-06	0.049	Subtree 1	S3’-desacetyl-phyllanthoside
342443	ITGB1	0.61	5.9e-06	0.049	Subtree 1	S3’-desacetyl-phyllanthoside
328426	CLDN1	0.56	5.9e-06	0.049	Subtree 1	Phyllanthoside
844	ZNF823	0.65	5.0e-07	0.022	Subtree 2	Nesol
62794	FOXN4	-0.62	1.2e-06	0.030	Subtree 2	Beta carotene
327430	OGFOD2	-0.64	1.7e-06	0.035	Subtree 2	Resveratrol
90487	SDHC	0.57	3.6e-06	0.046	Subtree 2	Lupeol
643160	LCP1	-0.62	4.0e-06	0.046	Subtree 2	3-Bromo-4-dimethylamino-alpha-benzoyl cinnamonitrite
844	PBX4	0.60	4.2e-06	0.046	Subtree 2	Nesol
90487	PFKFB2	0.56	5.3e-06	0.048	Subtree 2	Lupeol
619043	KIR2DL2	-0.63	5.6e-06	0.049	Subtree 2	Phyllanthin
236613	SLC7A11	0.79	1.0e-13	0.000	Subtree 3	Plumbagin
32982	ATAD3B	-0.67	1.3e-08	0.003	Subtree 3	Curcumin
309909	PDCD11	-0.66	2.3e-08	0.005	Subtree 3	Nimbolide
32982	HNRNPR	-0.66	3.0e-08	0.005	Subtree 3	Curcumin
309909	RPL34P6	-0.64	1.0e-07	0.009	Subtree 3	Nimbolide
32982	RPL11	-0.64	1.0e-07	0.012	Subtree 3	Curcumin
32982	PNRC2	-0.64	1.0e-07	0.012	Subtree 3	Curcumin
236613	HDHD2	-0.63	1.0e-07	0.013	Subtree 3	Plumbagin
309909	RPL34	-0.63	1.0e-07	0.013	Subtree 3	Nimbolide
309909	HNRNPA1P55	-0.63	2.0e-07	0.014	Subtree 3	Nimbolide
87868	NOLC1	-0.68	2.0e-07	0.014	Subtree 3	Phenethyl mustard oil
87868	NPM3	-0.68	2.0e-07	0.014	Subtree 3	Phenethyl mustard oil
236613	NR2F1	0.62	2.0e-07	0.014	Subtree 3	Plumbagin
742021	ERICH1	-0.62	2.0e-07	0.017	Subtree 3	Curcumin tri adamantylaminoethylcarbonate
87868	SRPK1	-0.67	3.0e-07	0.018	Subtree 3	Phenethyl mustard oil
309909	RPS10P2	-0.62	3.0e-07	0.018	Subtree 3	Nimbolide
87868	RBMXP1	-0.67	3.0e-07	0.018	Subtree 3	Phenethyl mustard oil
742020	RPL21P134	-0.61	3.0e-07	0.018	Subtree 3	Ethoxycurcumin trithiadiazolaminomethylcarbonte
705537	C5orf15	-0.63	4.0e-07	0.022	Subtree 3	Daturaolone
742022	CCDC149	0.61	4.0e-07	0.022	Subtree 3	Curcumin tri trithiadiazolaminoethylcarbonate
742020	RPL13AP3	-0.61	5.0e-07	0.022	Subtree 3	Ethoxycurcumin trithiadiazolaminomethylcarbonte
87868	ADAT2	-0.66	5.0e-07	0.022	Subtree 3	Phenethyl mustard oil
87868	HIF1A	0.66	5.0e-07	0.022	Subtree 3	Phenethyl mustard oil
236613	SLC7A11-AS1	0.60	5.0e-07	0.022	Subtree 3	Plumbagin
32982	SPEN	-0.60	7.0e-07	0.027	Subtree 3	Curcumin
236613	ACTN4P1	0.60	7.0e-07	0.027	Subtree 3	Plumbagin
643769	SMARCC1	-0.65	7.0e-07	0.027	Subtree 3	O-Bromo-alpha-benzoyl cinnamonitrile
643769	RPSAP56	-0.65	7.0e-07	0.027	Subtree 3	O-Bromo-alpha-benzoyl cinnamonitrile
87868	RPL10AP2	-0.65	8.0e-07	0.027	Subtree 3	Phenethyl mustard oil
87868	HNRNPA1P64	-0.65	8.0e-07	0.027	Subtree 3	Phenethyl mustard oil
87868	RPL34P18	-0.65	8.0e-07	0.027	Subtree 3	Phenethyl mustard oil
32982	SRRM1	-0.60	8.0e-07	0.027	Subtree 3	Curcumin
643023	FBXL2	0.66	8.0e-07	0.027	Subtree 3	Alpha-Phenyl-2,5-dimethoxy-alpha-cinnamonitrile
742020	RSL24D1	-0.59	9.0e-07	0.027	Subtree 3	Ethoxycurcumin trithiadiazolaminomethylcarbonte
236613	G6PD	0.59	9.0e-07	0.027	Subtree 3	Plumbagin
87868	HNRNPA1P55	-0.65	1.0e-06	0.027	Subtree 3	Phenethyl mustard oil
309909	NPM3	-0.60	1.0e-06	0.027	Subtree 3	Nimbolide
742022	PFN4	0.59	1.0e-06	0.027	Subtree 3	Curcumin tri trithiadiazolaminoethylcarbonate
87868	RPS4XP8	-0.64	1.0e-06	0.027	Subtree 3	Phenethyl mustard oil
87868	RPS4XP1	-0.64	1.2e-06	0.030	Subtree 3	Phenethyl mustard oil
309909	RPS10P5	-0.59	1.2e-06	0.030	Subtree 3	Nimbolide
643023	ETNK2	0.65	1.4e-06	0.031	Subtree 3	Alpha-Phenyl-2,5-dimethoxy-alpha-cinnamonitrile
742020	HNRNPA1P4	-0.59	1.4e-06	0.031	Subtree 3	Ethoxycurcumin trithiadiazolaminomethylcarbonte
309909	HNRNPA1L2	-0.59	1.4e-06	0.031	Subtree 3	Nimbolide
742019	ITGAV	0.59	1.4e-06	0.031	Subtree 3	Ethoxycurcumin tribenzimidazolmethylcarbonte
742020	RPL27AP	-0.58	1.4e-06	0.031	Subtree 3	Ethoxycurcumin trithiadiazolaminomethylcarbonte
643769	RPSA	-0.64	1.5e-06	0.032	Subtree 3	O-Bromo-alpha-benzoyl cinnamonitrile
742019	RPL21P44	-0.58	1.5e-06	0.032	Subtree 3	Ethoxycurcumin tribenzimidazolmethylcarbonte
236613	SRXN1	0.58	1.7e-06	0.034	Subtree 3	Plumbagin
365798	TSKU	0.64	1.8e-06	0.036	Subtree 3	Piceatannol
705537	SGF29	0.60	1.8e-06	0.036	Subtree 3	Daturaolone
742022	PDCD11	-0.58	1.9e-06	0.036	Subtree 3	Curcumin tri trithiadiazolaminoethylcarbonate
87868	RPS4XP2	-0.63	1.9e-06	0.036	Subtree 3	Phenethyl mustard oil
32982	HDAC10	-0.58	1.9e-06	0.036	Subtree 3	Curcumin
742020	EEF1B2P1	-0.58	2.0e-06	0.037	Subtree 3	Ethoxycurcumin trithiadiazolaminomethylcarbonte
742019	LINC00472	0.58	2.0e-06	0.037	Subtree 3	Ethoxycurcumin tribenzimidazolmethylcarbonte
742021	HMBOX1	-0.58	2.2e-06	0.039	Subtree 3	Curcumin tri adamantylaminoethylcarbonate
309909	NOLC1	-0.58	2.2e-06	0.039	Subtree 3	Nimbolide
643023	REEP3	0.64	2.3e-06	0.040	Subtree 3	Alpha.-Phenyl-2,5-dimethoxy-alpha-cinnamonitrile
87868	RPS4XP19	-0.63	2.4e-06	0.040	Subtree 3	Phenethyl mustard oil
32982	NOC2L	-0.58	2.4e-06	0.041	Subtree 3	Curcumin
309909	RPL34P18	-0.58	2.4e-06	0.041	Subtree 3	Nimbolide
87868	MYC	-0.63	2.6e-06	0.043	Subtree 3	Phenethyl mustard oil
236613	ALDH3A2	0.57	2.7e-06	0.043	Subtree 3	Plumbagin
32982	RCC2P4	-0.58	2.7e-06	0.043	Subtree 3	Curcumin
32982	KHDRBS1	-0.58	2.8e-06	0.043	Subtree 3	Curcumin
32982	DFFB	-0.58	2.8e-06	0.043	Subtree 3	Curcumin
87868	HNRNPA1P35	-0.62	3.0e-06	0.043	Subtree 3	Phenethyl mustard oil
643023	CAPN2	0.63	3.0e-06	0.043	Subtree 3	Alpha.-Phenyl-2,5-dimethoxy-alpha-cinnamonitrile
309909	ANLN	0.57	3.0e-06	0.043	Subtree 3	Nimbolide
32982	AHNAK2	0.57	3.1e-06	0.043	Subtree 3	Curcumin
236613	LOC344887	0.57	3.1e-06	0.043	Subtree 3	Plumbagin
309909	HNRNPA1P64	-0.57	3.2e-06	0.043	Subtree 3	Nimbolide
309909	EIF4BP9	-0.57	3.2e-06	0.043	Subtree 3	Nimbolide
309909	RPL29P7	-0.57	3.2e-06	0.043	Subtree 3	Nimbolide
742022	LOC100128816	-0.57	3.2e-06	0.043	Subtree 3	Curcumin tri trithiadiazolaminoethylcarbonate
742022	CUEDC1	0.57	3.3e-06	0.044	Subtree 3	Curcumin tri trithiadiazolaminoethylcarbonate
32982	CLIP4	0.57	3.3e-06	0.045	Subtree 3	Curcumin
112166	CRKL	0.61	3.4e-06	0.045	Subtree 3	Cucurbitacin K
236613	PGRMC1	0.57	3.4e-06	0.045	Subtree 3	Plumbagin
742019	RPL21P12	-0.57	3.5e-06	0.046	Subtree 3	Ethoxycurcumin tribenzimidazolmethylcarbonte
32982	ATAD3A	-0.57	3.7e-06	0.046	Subtree 3	Curcumin
236613	LRRC8A	0.57	3.7e-06	0.046	Subtree 3	Plumbagin
236613	AFAP1	0.57	3.8e-06	0.046	Subtree 3	Plumbagin
742022	CAMSAP2	0.56	3.9e-06	0.046	Subtree 3	Curcumin tri trithiadiazolaminoethylcarbonate
236613	NEU3	-0.56	3.9e-06	0.046	Subtree 3	Plumbagin
742019	RPS11P1	-0.56	3.9e-06	0.046	Subtree 3	Ethoxycurcumin tribenzimidazolmethylcarbonte
643023	MT2P1	0.63	4.1e-06	0.046	Subtree 3	Alpha-Phenyl-2,5-dimethoxy-alpha-cinnamonitrile
309909	ACTN4P1	0.57	4.1e-06	0.046	Subtree 3	Nimbolide
309909	IKZF5	-0.57	4.2e-06	0.046	Subtree 3	Nimbolide
87868	RPL34P31	-0.61	4.2e-06	0.046	Subtree 3	Phenethyl mustard oil
742019	FIGN	0.56	4.3e-06	0.046	Subtree 3	Ethoxycurcumin tribenzimidazolmethylcarbonte
236613	ELP2	-0.56	4.4e-06	0.046	Subtree 3	Plumbagin
309909	HNRNPCP3	-0.57	4.4e-06	0.046	Subtree 3	Nimbolide
643769	RPSAP47	-0.61	4.4e-06	0.046	Subtree 3	O-Bromo-alpha-benzoyl cinnamonitrile
309909	MTPAP	-0.57	4.4e-06	0.046	Subtree 3	Nimbolide
742020	RPL21P120	-0.56	4.5e-06	0.046	Subtree 3	Ethoxycurcumin trithiadiazolaminomethylcarbonte
742022	CRACR2A	-0.56	4.5e-06	0.046	Subtree 3	Curcumin tri trithiadiazolaminoethylcarbonate
236613	NQO1	0.56	4.5e-06	0.046	Subtree 3	Plumbagin
643769	RPS10	-0.61	4.5e-06	0.046	Subtree 3	O-Bromo-alpha-benzoyl cinnamonitrile
705537	PDGFC	-0.58	4.6e-06	0.046	Subtree 3	Daturaolone
742022	DARS	-0.56	4.6e-06	0.046	Subtree 3	Curcumin tri trithiadiazolaminoethylcarbonate
236613	ANXA2P1	0.56	4.7e-06	0.046	Subtree 3	Plumbagin
309909	EIF4BP5	-0.57	4.7e-06	0.046	Subtree 3	Nimbolide
87868	PDSS1	-0.61	4.7e-06	0.046	Subtree 3	Phenethyl mustard oil
309909	RPL7AP12	-0.56	4.8e-06	0.046	Subtree 3	Nimbolide
383468	RCC2P4	-0.56	4.8e-06	0.046	Subtree 3	Andrographis Paniculata
32982	RASAL2	0.56	4.8e-06	0.046	Subtree 3	Curcumin
309909	RPL34P31	-0.56	5.0e-06	0.047	Subtree 3	Nimbolide
87868	COL4A1	0.61	5.0e-06	0.047	Subtree 3	Phenethyl mustard oil
309909	SFXN2	-0.56	5.0e-06	0.047	Subtree 3	Nimbolide
643769	HNRNPM	-0.61	5.2e-06	0.048	Subtree 3	O-Bromo-alpha-benzoyl cinnamonitrile
87868	PDGFD	0.61	5.2e-06	0.048	Subtree 3	Phenethyl mustard oil
87868	RPL36AP39	-0.61	5.2e-06	0.048	Subtree 3	Phenethyl mustard oil
87868	HNRNPA1P13	-0.61	5.3e-06	0.048	Subtree 3	Phenethyl mustard oil
309909	SNORA14B	-0.56	5.3e-06	0.048	Subtree 3	Nimbolide
87868	BICC1	0.61	5.4e-06	0.048	Subtree 3	Phenethyl mustard oil
87868	PRSS23	0.61	5.4e-06	0.048	Subtree 3	Phenethyl mustard oil
742022	RPL6	-0.56	5.5e-06	0.048	Subtree 3	curcumin tri trithiadiazolaminoethylcarbonate
87868	RPL34P6	-0.61	5.5e-06	0.048	Subtree 3	Phenethyl mustard oil
87868	HNRNPA1P8	-0.61	5.5e-06	0.048	Subtree 3	Phenethyl mustard oil
309909	TAF5	-0.56	5.6e-06	0.048	Subtree 3	Nimbolide
32982	PRTG	0.56	5.6e-06	0.048	Subtree 3	Curcumin
643023	ITGA3	0.62	5.7e-06	0.049	Subtree 3	Alpha.-Phenyl-2,5-dimethoxy-alpha-cinnamonitrile
643769	CTSD	0.61	5.7e-06	0.049	Subtree 3	O-Bromo-alpha-benzoyl cinnamonitrile
309909	GALNT10	0.56	5.8e-06	0.049	Subtree 3	Nimbolide
742020	TFAP4	-0.56	6.0e-06	0.050	Subtree 3	Ethoxycurcumin trithiadiazolaminomethylcarbonte
309909	RPL10AP2	-0.56	6.0e-06	0.050	Subtree 3	Nimbolide

Listed are the genes whose expression was associated with logGI50 of INPs with FDR adjusted *p* < 0.05. For each product, the subtree from hierarchical clustering shown in Fig. 1 is provided. The product of *Ricinus communis* (NSC 15384) was not included in the hierarchical clustering as its screening concentration units differed from all other INPs. ρ, Spearman correlation coefficient

### SLC7A11 and plumbagin (NSC 688284)

SLC7A11 (solute carrier family 7 member 11) has recently been suggested as potential drug target in pancreatic adenocarcinoma [37]. It plays a role in maintaining cellular glutathione levels via cystine uptake, protecting cells from oxidative stress induced death and is commonly overexpressed in cancer, which has been linked to chemoresistance in many anti-tumor agents [38–41]. Deletion of the SLC7A11 gene in genetically engineered mice with pancreatic ductal adenocarcinoma induced tumor-selective ferroptosis and inhibited tumor growth [40]. Targeting of the SLC7A11/glutathione axis with sulfasalazine has been shown to cause synthetic lethality via decreased cystine uptake and intracellular glutathione biosynthesis [42]. Alternative strategies leveraging this metabolic addiction have also been demonstrated via inhibiting glucose uptake preventing the conversion of potentially toxic cystine to cysteine [38, 43]. This highly positive correlation (Spearman correlation coefficient *ρ* = 0.79, unadjusted *p* value = 1.07 × 10^–13^, FDR adjusted *p* value = 8.47 × 10^–8^) demonstrates increased resistance of tumor cell lines to plumbagin associated with increased gene expression of SLC7A11, which is consistent with the previous findings by our group and other authors about the potential role of this transporter in resistance to multiple antitumor agents and natural products [29, 38, 42, 44].

### ATAD family and curcumin

ATAD3A and ATAD3B are mitochondrial ATPase proteins expressed in embryogenesis. ATAD3B has been shown to be over-expressed in head and neck cancer and hepatocellular carcinoma [45, 46]. Curcumin acts as a protonorphic uncoupler of oxidative phosphorylation decreasing ATP biosynthesis which alters the AMP:ATP ratio and ultimately decreases cell proliferation [47]. The negative correlation for both ATAD3A (Spearman correlation coefficient *ρ* = -0.57, unadjusted *p* value = 3.68 × 10^–6^, FDR adjusted *p* value = 0.04) and ATAD3B (Spearman *ρ* = -0.67, unadjusted *p* value = 1.29 × 10^–8^, FDR adjusted *p* value = 3.4 × 10^–3^) genes demonstrates that increased sensitivity of cell lines to curcumin (i.e., lower logGI50 values) was associated with increased expression of the ATAD3A and ATAD3B genes.

### MYB and phyllanthoside

MYB, a transcriptional activator, is a proto-oncogene that has been shown to be over-expressed in hematologic, colorectal, and breast cancer [48]. The negative correlation (Spearman correlation coefficient *ρ* = -0.66, unadjusted p value = 1.69 × 10^–8^, FDR adjusted *p* value = 3.84 × 10^–3^) demonstrates an association between increased sensitivity of cell lines to phyllanthoside and increased expression of the MYB gene. This suggests a potential role of MYB-mediated transcriptional regulation in response to this INP.

### Biological pathway analysis

The results of pathway analysis using g:Profiler are presented in Supplementary Tables 1–3 and summarized in a graphical format in Supplementary Fig. 5. Below we discuss the pathways and molecular functions that were identified for Subtrees 1 and 3. Subtree 2 was not evaluable due to a paucity of significant genes.

Biological pathway analysis using g:Profiler identified several biological pathways and functions which may be associated with increased sensitivity or resistance to INPs. Among the INPs in Subtree 1, resistance to NSC number 328426 (phyllanthoside), 342443 (S3’-desacetyl-phyllanthoside), 94743 (cucurbitacin A), 143925 (pekilocerin A), 112167 (elatericin B) was associated with pathways related to mineral homeostasis (Supplementary Table 1). Due to an insufficient number of genes associated with sensitivity to INPs in Subtree 1, common biological processes for those genes and INPs could not be evaluated.

### Subtree 3

Among the INPs in Subtree 3, response to NSC number 236613 (plumbagin), 643023 (alpha-phenyl-2,5-dimethoxy-alpha-cinnamonitrile), 365798 (piceatannol), 112166 (cucurbitacin K) and sensitivity to 32982 (curcumin), 309909 (nimbolide), 87868 (phenethyl mustard oil), 742021 (curcumin tri adamantylaminoethylcarbonate), 742019 (ethoxycurcumin trithiadiazolaminomethylcarbonte), 705537 (daturaolone), 643769 (O-bromo-alpha-benzoyl cinnamonitrile), 383468 (product of *Andrographis paniculata*) was associated with expression of genes involved in several molecular pathways (Supplementary Tables 2 and 3). Molecular functions associated with drug response in Subtree 3 include nucleic acid binding, heterocyclic compound binding, organic cyclic compound binding, and multiple aspects of protein synthesis including various stages of translation and structural components of the ribosome.

### Nuclear factor erythroid 2-related factor 2 (NRF2) pathway

NRF2 is a key transcription factor and a key modulator of cellular antioxidant response which has a role in preventing carcinogenesis. However, persistent activation of NRF2 has been demonstrated in some tumor types, which raises a possibility of its role in cancer proliferation [49]. As expression of the genes in this pathway was positively correlated with the INPs in Subtree 3, this suggests that resistance mechanisms to these INPs may be related to the NRF2 pathway [50].

### PI3K-Akt-mTOR pathway

Overactivation of the PI3K-Akt-mTOR signaling pathway has been demonstrated in many different cancer types as a mechanism for tumor growth and therapeutic resistance [51]. As the pathway analysis of expression of the genes in this pathway found a positive correlation with logGI50 of the INPs in Subtree 3, this suggests that resistance mechanisms to the INPs such as NSC number 236613 (plumbagin), 643023 (alpha-phenyl-2,5-dimethoxy-alpha-cinnamonitrile), 365798 (piceatannol) and 112166 (cucurbitacin K) may be related to the PI3K-Akt-mTOR signaling. Subtree 3 contained several curcumin INPs and gallocatechin, which have been previously demonstrated to be associated with this pathway [52].

### Eukaryotic translation pathway

A crucial component of cancer progression is translational control of protein synthesis through a increased rates of protein synthesis and specific mRNAs that promote increased tumor cell growth and survival [53]. As the pathway analysis of expression of genes in this pathway found a negative correlation with logGI50 of the INPs in Subtree 3, this suggests that sensitivity mechanisms to these INPs may be related to pathways associated with protein synthesis inhibition. Subtree 3 contained several curcumin-related INPs which have been previously demonstrated to have an association with these pathways [54].

### Slit/Robo pathway

While the Slit/Robo pathway mainly involves functions to promote axon branching and neuronal migration, it is also involved in other physiological processes including angiogenesis and apoptosis [55]. Promoter hypermethylation of Slit/Robo has been observed in many different cancers, leading to undetectable or low levels of Slit/Robo, and natural products that reactivate this pathway via demethylation or other mechanisms are actively being explored [55]. Increased expression of genes in this pathway was negatively correlated with logGI50 of several INPs in Subtree 3, including NSC number 32982 (curcumin), 309909 (nimbolide), 87868 (phenethyl mustard oil), 742021 (curcumin tri adamantylaminoethylcarbonate), 742020 (ethoxycurcumin trithiadiazolaminomethylcarbonte), 705537 (daturaolone), 643769 (O-bromo-alpha-benzoyl cinnamonitrile), and 383468 (product of *Andrographis paniculata*), suggesting that overexpression of those genes may confer increased sensitivity to these products. This association indicates that such INPs could be explored to target this pathway. Curcumin and its related analogues have been demonstrated to also have a demethylating effect [56].

### Association of cell line response to INPs with protein-changing single nucleotide variants

For each of the 75 INPs, and using whole exome sequencing data for the cell lines from CellMiner after filtering, we used a Student’s *t*-test to analyze the differences between logGI50 values comparing cell lines with and without individual protein-changing single nucleotide variants in each of the 107 genes listed in OncoKB. After FDR adjustment, 13 SNV-INP pairs satisfied the FDR adjusted *p* value < 0.05, including 4 unique genes and 10 unique natural products. Below we discuss examples of associations of functionally important variants and likely oncogenic variants from OncoKB (Table 3 and Supplementary Fig. 6).

**Table 3:** Association of functionally important variants and likely oncogenic variants with response to Indian natural products

NSC number	Gene	Variant	*p*-value	*t*-statistic	# of cell lines with variant	# of cell lines without variant	Mean logGI50 with variant	Mean logGI50 without variant	Prevalence in 1000 Genomes	SNP Type	OncoKB level	OncoKB annotation	FDR adjusted *p*-value	INP name
112166	MET	T992I	0.0000368	-4.782802	3	47	-5.806500	-5.703000	0.01	Missense	NA	Likely Oncogenic	0.0027367	Cucurbitacin K
112167	MET	T992I	0.0006802	-3.743424	3	47	-6.801167	-6.709511	0.01	Missense	NA	Likely Oncogenic	0.0243927	Elatericin B
308606	BRAF	V600E	0.0000006	-6.134195	9	41	-7.159667	-6.693878	0.00	Missense	1	Oncogenic	0.0000742	Cucurbitacin D
328426	KDR	C482R	0.0019152	-3.308867	3	56	-8.222667	-7.909268	0.01	Missense	NA	Likely Oncogenic	0.0414968	Phyllanthoside
643160	MET	T992I	0.0001938	4.076171	3	44	-4.000000	-4.145977	0.01	Missense	NA	Likely Oncogenic	0.0089764	3-Bromo-4-dimethylamino-alpha-benzoyl cinnamonitrite
710351	KDR	C482R	0.0017855	3.286695	3	55	-4.000000	-4.137891	0.01	Missense	NA	Likely Oncogenic	0.0406327	Achilleol A
710351	MET	T992I	0.0017855	3.286695	3	55	-4.000000	-4.137891	0.01	Missense	NA	Likely Oncogenic	0.0406327	Achilleol A
717335	MET	T992I	0.0000616	-4.338376	3	55	-7.993333	-7.741146	0.01	Missense	NA	Likely Oncogenic	0.0042724	Tylophorin
717335	KNSTRN	A40E	0.0017579	-3.300053	7	51	-7.954286	-7.726725	0.06	Missense	NA	Likely Oncogenic	0.0406327	Tylophorin
731920	KNSTRN	A40E	0.0007237	-3.790673	7	42	-4.671286	-4.511429	0.06	Missense	NA	Likely Oncogenic	0.0250871	Calendulaglycoside B-6'-O-butyl ester
731921	KDR	C482R	0.0014349	-3.902824	3	48	-5.905667	-5.644375	0.01	Missense	NA	Likely Oncogenic	0.0406327	Calendulaglycoside D2
731922	KDR	C482R	0.0017287	-3.535183	3	47	-5.191333	-5.088383	0.01	Missense	NA	Likely Oncogenic	0.0406327	Calendulaglycoside D-6'-O-methyl ester
731922	KNSTRN	A40E	0.0010801	-3.482230	7	43	-5.183857	-5.080023	0.06	Missense	NA	Likely Oncogenic	0.0351046	Calendulaglycoside D-6'-O-methyl ester

*NSC* INP NSC number, *Gene* Gene name, *Variant* Sequence variant, *p*-*value* original *p*-value (prior to adjustment for multiple testing) from the Student’s *t-*test comparing the mean logGI50 values in those cell lines that had each variant to those that were not reported to have the variant, *t*-statistic value from the Student’s *t-*test comparing the mean logGI50 values in those cell lines that had each variant to those that were not reported to have the variant, **# ***of cell lines with variant* Number of NCI-60 cell lines which had that variant according to information from CellMiner, **# ***of cell lines without variant* number of NCI-60 cell lines which were not reported to have that variant according to data from CellMiner, *Mean logGI50 with variant* Average logGI50 value in NCI-60 cell lines that had the variant, *Mean logGI50 without variant* average logGI50 value in NCI-60 cell lines not reported to have the variant, *Prevalence in 1000 Genomes* Frequency of the variant in the 1000 Genomes dataset, according to CellMiner, *OncoKB level* Highest level of evidence for the variant across tissues according to the OncoKB annotation; *OncoKB annotation*, OncoKB classification as oncogenic or likely oncogenic, *FDR adjusted* *p*-value, *p*-value (adjusted for multiple testing) from the Student’s *t-*test comparing the mean logGI50 values in those cell lines that had each variant to those that were not reported to have the variant; *INP name*, name of the Indian natural product

### BRAF V600E and Cucurbitacin D (NSC 308606)

OncoKB lists BRAF V600E as a level 1 actionable variant, which was present in 9 cell lines (7 melanoma and 2 colorectal cell lines) in the NCI-60 dataset. Tumors with this variant are responsive to treatment with BRAF inhibitors (e.g., dabrafenib, vemurafenib) and in combination with MEK inhibitors this has been shown to be an effective treatment strategy for melanoma [57]. Consistent with our earlier analysis of a separate large natural product dataset [29], mean logGI50 response to cucurbitacin D was statistically significantly different when comparing cell lines without the BRAF V600E variant (mean = -6.69) to those with this variant (mean = -7.16, unadjusted *p* value = 5.71 × 10^–7^; FDR adjusted *p* value = 7.42 × 10^–5^). This association suggests that cucurbitacin D may have a role in targeting cancers with BRAF mutations or having an effect on BRAF [58]. Alternatively, the presence of BRAF V600E in most of the melanoma lines (8 out of 9 melanoma cell lines) may suggest that this INP may have a more general effect on growth inhibition in melanoma.

### Likely oncogenic or likely gain of function variants

Multiple INPs were significantly associated with likely oncogenic individual variants listed in OncoKB in the KDR and KNSTRN genes (C482R and A40E, respectively) and the likely gain of function variant T992I in MET.

The receptor tyrosine kinase MET gene variant T992I was associated with sensitivity to multiple INPs, including products from the cucurbitacin family (Curcurbitacin K; NSC 112166, Elatericin B; NSC 112167) and the Tylophorine family (tylophorin, NSC 717335) and resistance to other products (3-bromo-4-dimethylamino-.alpha.-benzoyl cinnamonitrite; NSC 643160, achilleol A; NSC 710351).

The likely oncogenic, likely gain of function KDR gene variant C482R was associated with sensitivity to two INPs from the *Calendula* family (calendulaglycoside D2; NSC 731921, calendulaglycoside D-6'-O-methyl ester; NSC 731922) and the *Phyllanthus* family (phyllathoside, NSC 328426) and resistance to achilleol A (NSC 710351).

The likely oncogenic, likely gain of function kinetochore KNSTRN gene variant A40E was associated with sensitivity to three INPs (tylophorin; NSC 717335, calendulaglycoside B-6'-O-butyl ester; NSC 731920 and calendulaglycoside D-6'-O-methyl ester; NSC 731922).

## Discussion

In this study, we used in vitro data to examine the associations of variation in gene expression and deleterious mutations with tumor cell response to INPs. We also compared response patterns to those of reference compounds as a preliminary investigation of the possible mechanisms of action of these products at the cellular level. We reported the findings that were highly significant after the correction for multiple comparisons. We compared publicly available cancer cell line response data in the NCI-60 panel for 75 INPs to data for standard reference antitumor compounds. Our joint analysis of molecular data and measures of cell line response to INPs and the comparison of the cytotoxic effects of INPs to those of established antitumor reference compounds allowed us to quantitively assess the potential involvement of individual genes and molecular pathways in tumor cell response to INPs. In Supplementary Figs. 4–6, we provide the summary of significant associations between the logGI50 measures of cancer cell line response to 75 INPs and molecular features of the tumor cells including gene expression, biological pathways, and single nucleotide variants in cancer-related genes.

Subtree 1 from the clustering of logGI50 values of INPs and reference compounds consisted of many products with anti-mitotic mechanisms of action, confirming previously reporting anti-mitotic activity of some INPs including phyllanthoside, S3’-desacetyl-phyllanthoside and the cucurbitacin family[13, 34]. Overall, the logGI50 response data were closely grouped among similar products, including cucurbitacins in Subtree 1, and curcumin and curcuminoids in Subtree 3.

Our analysis found multiple novel associations between gene expression and logGI50 values of INPs, including a highly significant association between increased levels of SLC7A11 expression and resistance to plumbagin. This resistance may involve increased SLC7A11 expression inhibiting ferroptosis, a distinct form of cell death due to excessive lipid peroxidation [43]. To our knowledge, our observed association between increased levels of ATAD3A*/* ATAD3B expression and sensitivity to curcumin has not been previously reported. The products of these genes, ATPase family AAA domain containing 3A and 3B proteins, are involved in multi-protein complexes associated with mtDNA that are important for regulation of mitochondrial biogenesis and lipogenesis. Curcumin has been reported to regulate expression of enzymes involved in mitochondrial biogenesis and mitochondrial oxidative stress, to increase apoptosis and autophagic cell death, and to reduce cellular proliferation [59–62]. The association with ATAD3A and ATAD3B expression may be of interest since ATAD3 over-expression has been linked to the progression of head and neck cancer, lung adenocarcinoma, non‑Hodgkin's lymphoma, uterine cancer, cervical cancer, prostate cancer, glioma, and hepatocellular carcinoma [46, 63, 64]. Interestingly, prior reports suggested the roles of increased ATAD3 expression in chemoresistance [46].

Our analysis of SNV variants demonstrated a statistically significant association of BRAF V600E with logGI50 measure of response to cucurbitacin D. The triterpene compounds from the *Cucurbitaceae* family, which include cucurbitacin D, are found in many gourd species. While they have demonstrated cytotoxicity in many cell lines, our finding of increased sensitivity in BRAF V600E mutated cell lines which includes almost all the melanoma cell lines in our dataset may warrant further investigation.

Paucity of INPs available in the public domain and consequently their underrepresentation in the NCI-60 cell line database limited our ability to evaluate some of the more commonly used Ayurvedic concoctions and herbs of interest including *Triphala, Momordica charantia*, and *Withania somnifera*. Additional open-source natural products databases [65–67] contain more INPs; however, the available NCI-60 screening data for these additional products in the DTP dataset were limited to single dose data and were not analyzed in our study.

We used logGI50 values as the primary response endpoint because many previous studies have shown these measures to be a relevant outcome to study associations with molecular targets. When using logGI50 values, clusters of compounds derived from logGI50 values have been shown to correlate well both with potential mechanism of cell line response and with similarities among compound structures [18, 28, 29, 68–70].

We used median logGI50 derived from the five-range dose screen as our measure of cell line response for the analysis of associations with molecular features of tumor cell lines. While this single logGI50 measure is informative in characterizing the cytotoxic effect of individual products, it may not reflect the cytotoxicity of the compound if it fell outside the pre-defined range of activity, in which case this measure would not reflect low levels of activity of some of the compounds we analyzed. As we analyzed pre-treatment gene expression levels for each cancer cell line, our findings cannot characterize the association between cell line response and post-treatment gene expression changes in response to each INP or reference compound. Such analyses may be of potential benefit in the future if post-treatment response data for Indian natural products become available. As the NCI-60 panel does not include normal cell lines for comparison, we did not focus on toxicity of these compounds and further studies will need to examine the side effects of these INPs.

As a note of caution, our findings do not indicate clinical efficacy but rather our study is an attempt to characterize available INPs and identify possible mechanisms of action for further study. In this analysis, utilization of the in vitro molecular screening data from the NCI-60 allowed us to identify molecular features of tumor cells associated with response to INPs. As Ayurvedic products are often used in specific combinations, our analysis would not be able to evaluate their clinical and immunomodulatory features associated with response to the combinations of such agents. Additionally, due to the limited representation of tumors and mutational features in the NCI-60 panel, we could not examine the response within individual cancer categories. Additional models including mouse patient-derived xenografts or other clinically relevant approaches may be needed to further investigate the physiological effects of Ayurvedic products in specific tumor types.

## Conclusions

Our analysis examining NCI60 response patterns for 75 INPs and standard reference compounds and their similarities allowed us to elucidate potential common mechanisms of action and molecular features associated with response to these INPs. We identified a number of genes and several biological pathways that were associated with sensitivity and resistance to specific INPs and/or entire INP clusters. Our findings provide a proof of principle that INPs may represent compounds of interest for cancer drug discovery and further studies should increase our understanding of their possible mechanisms of action.

## Supplementary Information

**Additional file 1.** Supplementary Figure 1. Heatmap of median logGI50 values of Indian natural products and reference compounds. Each row represents an Indian natural product or a standard reference compound and each column represents a cell line in the NCI-60 cancer cell line panel. The color key represents the logGI50 levels with negative values (blue) representing sensitivity of a cell line to the product and positive values (red) representing resistance to a product. Missing data are represented as black. The range of logGI50 values was -12.5 to -0.25 molar units.

**Additional file 2.** Supplementary Figure 2. Hierarchical clustering of INPs and reference compounds based on their median logLC50 values across NCI60 cell lines. The tree was inferred using the UPGMA (‘average’) method and was based on Euclidean distances. The tree is presented as an unrooted radial phylogram. The scale in the top left corner is provided for the branch lengths, which were derived from Euclidean distances. Clustered products are displayed with sparse labeling, in which only a random subset of INP labels is displayed.

**Additional file 3. **Supplementary Figure 3. Hierarchical clustering of INPs and reference compounds based on their median total growth inhibition (TGI) values across NCI60 cell lines. The tree was inferred using the UPGMA (‘average’) method and was based on Euclidean distances. The tree is presented as an unrooted radial phylogram. The scale in the top left corner is provided for the branch lengths, which were derived from Euclidean distances. Clustered products are displayed with sparse labeling, in which only a random subset of INP labels is displayed.

**Additional file 4. **Supplementary Figure 4. Graphical overview of significant associations logGI50 of Indian natural products with gene expression. Shown are significant associations with FDR adjusted *p* < 0.05, which are listed in Table 2. INPs are presented by colored circles, with colors corresponding to their subtree assignment based on clustering of their logGI50 values (orange for subtree 1, red for subtree 2, and purple for subtree 3). The subtree assignment of the INPs based on the logGI50 values is shown in Fig. 1, Supplementary Fig. 1, Table 1, and Supplementary Table 5. The direction of the arrows corresponds to the negative or positive values of the Spearman correlation coefficient ρ of association between gene expression and logGI50. An arrow toward an INP indicates ρ > 0, when higher gene expression was associated with higher logGI50 values and increased cell line resistance to that INP, whereas an arrow toward a gene indicates ρ < 0, showing that higher gene expression was associated with lower logGI50 values and with increased cell line sensitivity to that INP.

**Additional file 5.** Supplementary Figure 5. Graphical overview of significant associations of logGI50 of Indian natural product subtrees 1 and 3 with molecular pathways from Reactome, KEGG, and WikiPathways. Shown are significant associations identified by g:Profiler with FDR adjusted *p* < 0.05. **(A) **Positive associations for Subtree 1. **(B) **Positive associations for Subtree 3. **(C) **Negative associations for Subtree 3. Additional information about each association shown in the Figure is provided in Supplementary Tables 1-3.

**Additional file 6.** Supplementary Figure 6. Graphical overview of significant associations of logGI50 of Indian natural products with protein-changing SNVs in cancer-related genes, which are listed in Table 3. Shown are significant associations with FDR adjusted *p* < 0.05. INPs are presented by colored circles, with colors corresponding to their subtree assignment based on clustering of their logGI50 values shown in Fig. 1, Supplementary Fig. 1, and Table 1 (orange for subtree 1, red for subtree 2, and purple for subtree 3). The direction of the arrows corresponds to the negative or positive values of the *t*-statistic in the Student’s *t*-test. An arrow toward an INP indicates a positive value of the *t*-statistic, suggesting increased cell line resistance to that INP in the presence of a variant. In contrast, an arrow toward a variant indicates a negative value of the *t*-statistic, suggesting increased cell line sensitivity to that INP in the presence of a variant.

**Additional file 7.** Supplementary Table 1: Positively correlated pathways in Subtree 1

**Additional file 8.** Supplementary Table 2: Positively correlated pathways in Subtree 3

**Additional file 9.** Supplementary Table 3: Negatively correlated pathways in Subtree 3

**Additional file 10.** Supplementary Table 4: All queried Ayurvedic INPs from the PUBLIC COMPARE portal

**Additional file 11.** Supplementary Table 5: Concordance between the clustering of Indian natural products and reference compounds based on logGI50, logLC50, and TGI values

## Data Availability

All response data for the INPs and the reference compounds used in this analysis are publicly available at the DTP PUBLIC COMPARE portal (https://dtp.cancer.gov/public_compare) [24, 25]. NCI-60 expression and single nucleotide variant data are publicly available from the CellMiner and CellMinerCDB online resources [22, 28].

## Abbreviations

ROS: Reactive oxygen species
SNVs: Single nucleotide variants
NCI: National Cancer Institute
BRP: Biometric Research Program
TGI: Total growth inhibition
FDR: False discovery rate
WES: Whole exome sequencing
SLC7A11: Solute carrier family 7 member 11
NRF2: Nuclear factor erythroid 2-related factor 2
PDX: Patient-derived xenograft
