# Supplementary figures and images for: Pharmacogenomics of in vitro response of the NCI-60 cancer cell line panel to Indian natural products
Source: BMC Cancer. 2022 May 7;22:512. doi: 10.1186/s12885-022-09580-7 (PMC9077913; doi:10.1186/s12885-022-09580-7)

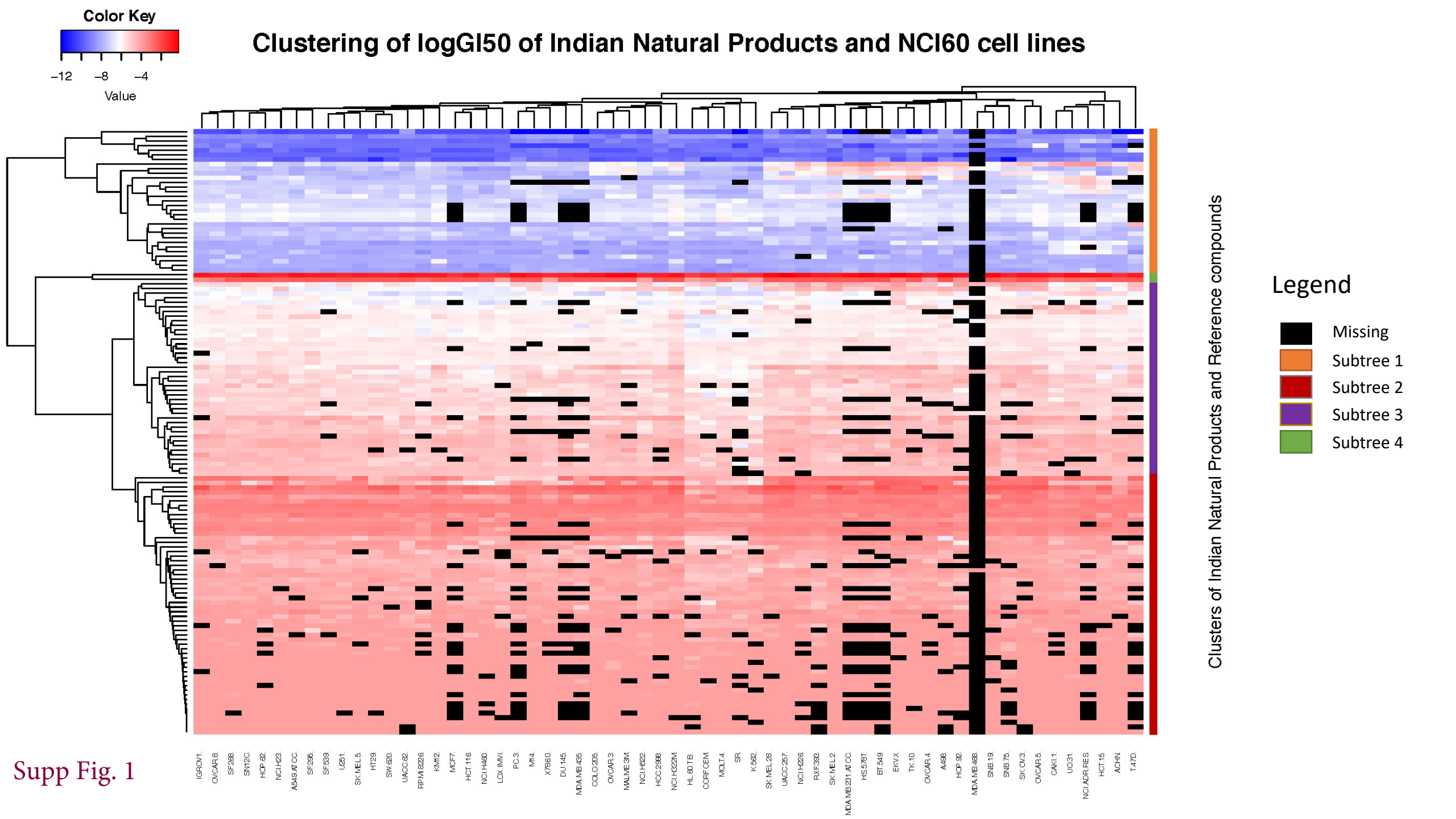

Supp Fig. 1

Supplement: Supplementary file 1 — Additional file 1. Supplementary Figure 1. Heatmap of median logGI50 values of Indian natural products and reference compounds. Each row represents an Indian natural product or a standard reference compound and each column represents a cell line in the NCI-60 cancer cell line panel. The color key represents the logGI50 levels with negative values (blue) representing sensitivity of a cell line to the product and positive values (red) representing resistance to a product. Missing data are represented as black. The range of logGI50 values was -12.5 to -0.25 molar units. [file 12885_2022_9580_MOESM1_ESM.pdf]

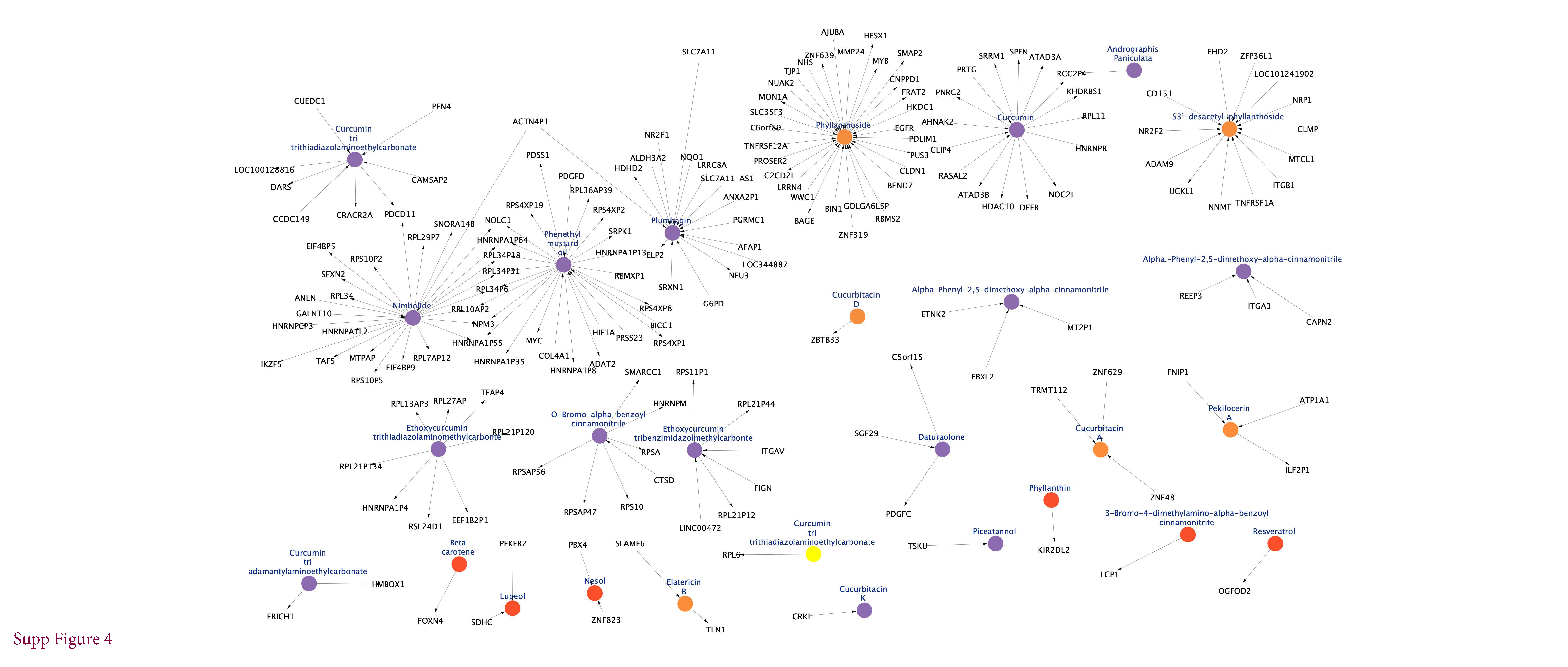

Supplement: Supplementary file 4 — Additional file 4. Supplementary Figure 4. Graphical overview of significant associations logGI50 of Indian natural products with gene expression. Shown are significant associations with FDR adjusted p < 0.05, which are listed in Table 2. INPs are presented by colored circles, with colors corresponding to their subtree assignment based on clustering of their logGI50 values (orange for subtree 1, red for subtree 2, and purple for subtree 3). The subtree assignment of the INPs based on the logGI50 values is shown in Fig. 1, Supplementary Fig. 1, Table 1, and Supplementary Table 5. The direction of the arrows corresponds to the negative or positive values of the Spearman correlation coefficient ρ of association between gene expression and logGI50. An arrow toward an INP indicates ρ > 0, when higher gene expression was associated with higher logGI50 values and increased cell line resistance to that INP, whereas an arrow toward a gene indicates ρ < 0, showing that higher gene expression was associated with lower logGI50 values and with increased cell line sensitivity to that INP. [file 12885_2022_9580_MOESM4_ESM.png]

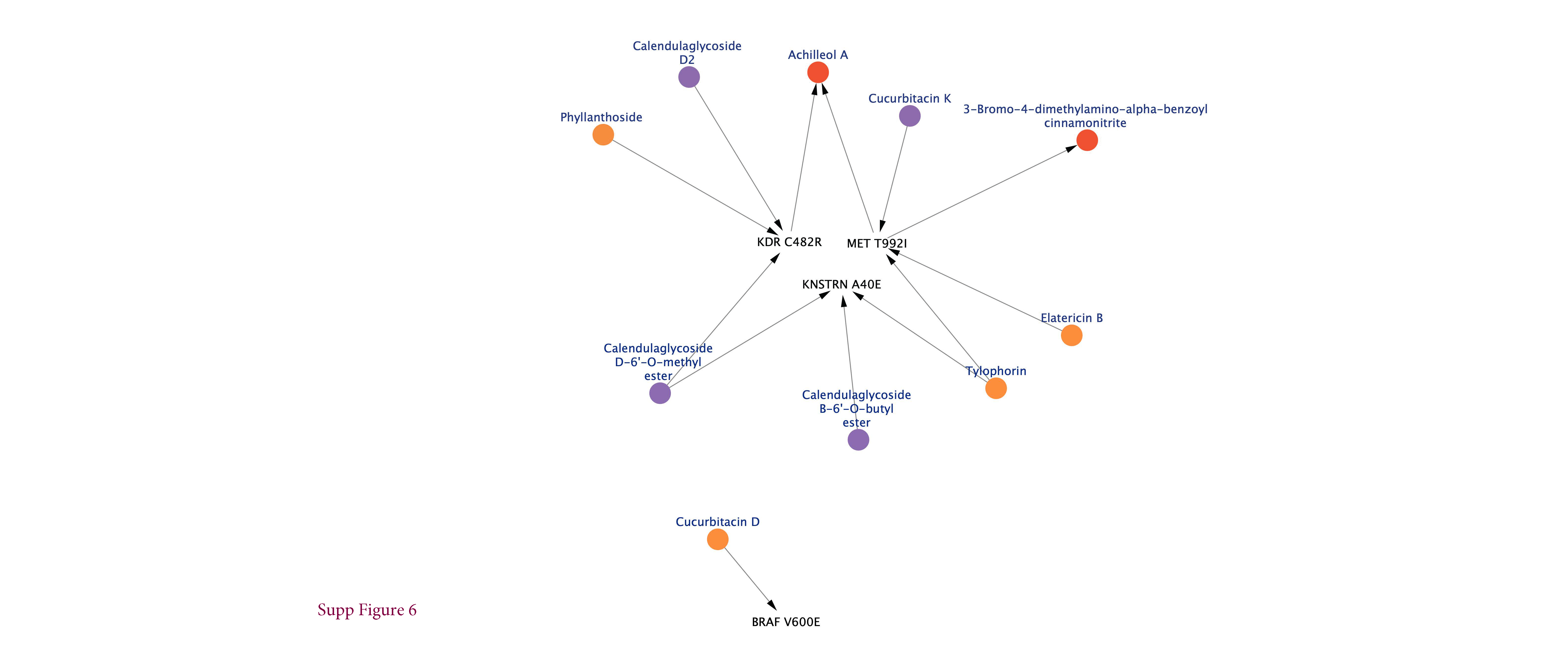

Supplement: Supplementary file 6 — Additional file 6. Supplementary Figure 6. Graphical overview of significant associations of logGI50 of Indian natural products with protein-changing SNVs in cancer-related genes, which are listed in Table 3. Shown are significant associations with FDR adjusted p < 0.05. INPs are presented by colored circles, with colors corresponding to their subtree assignment based on clustering of their logGI50 values shown in Fig. 1, Supplementary Fig. 1, and Table 1 (orange for subtree 1, red for subtree 2, and purple for subtree 3). The direction of the arrows corresponds to the negative or positive values of the t-statistic in the Student’s t-test. An arrow toward an INP indicates a positive value of the t-statistic, suggesting increased cell line resistance to that INP in the presence of a variant. In contrast, an arrow toward a variant indicates a negative value of the t-statistic, suggesting increased cell line sensitivity to that INP in the presence of a variant. [file 12885_2022_9580_MOESM6_ESM.png]
