# Supplementary material for: Pharmacogenomics of in vitro response of the NCI-60 cancer cell line panel to Indian natural products
Source: BMC Cancer. 2022 May 7;22:512. doi: 10.1186/s12885-022-09580-7 (PMC9077913; doi:10.1186/s12885-022-09580-7)

Supp Figure 2: Hierarchical clustering of INPs and reference compounds based on the median logLC50 values of the NCI-60 cell line panel

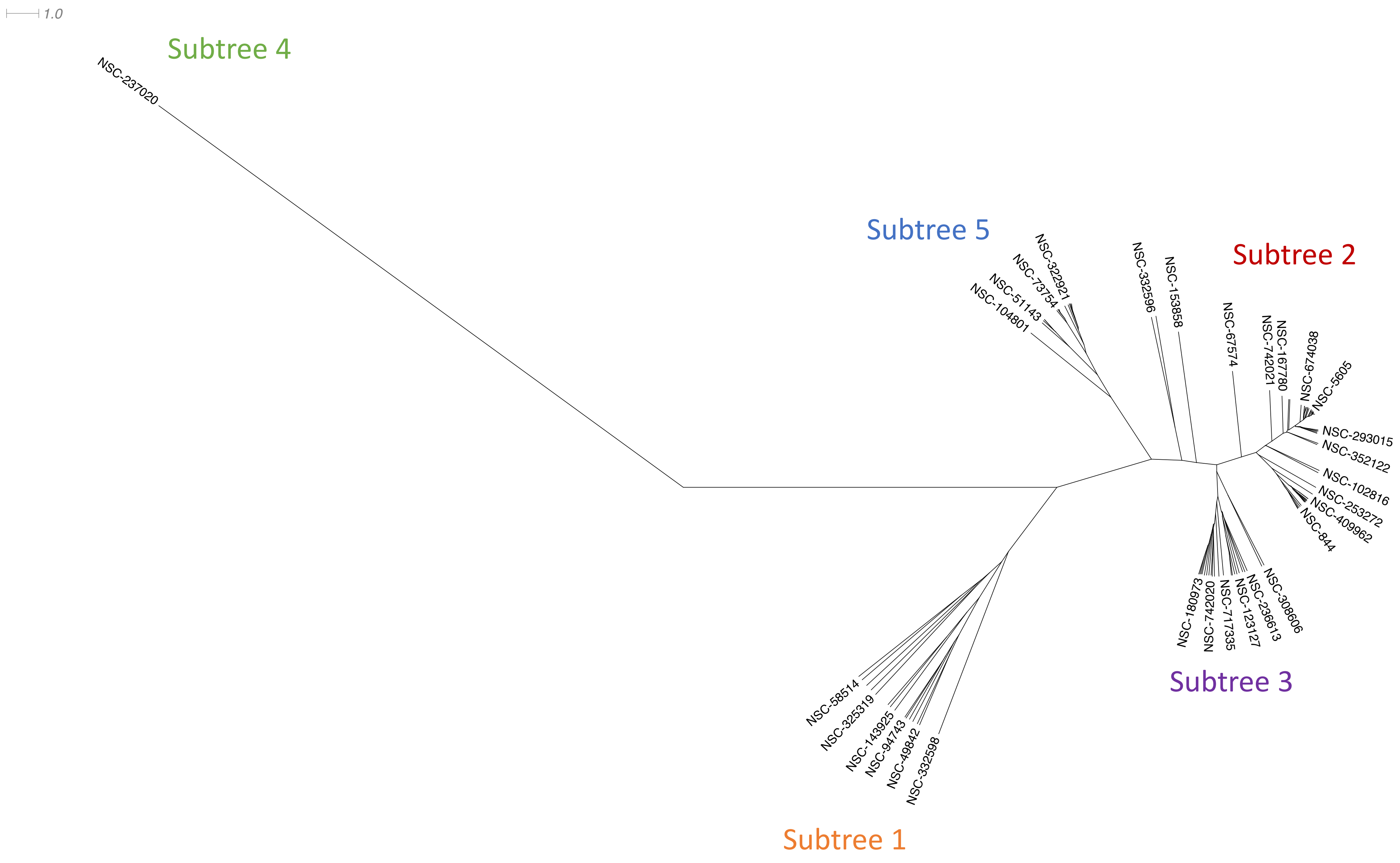

Supplement: Supplementary file 2 — Additional file 2. Supplementary Figure 2. Hierarchical clustering of INPs and reference compounds based on their median logLC50 values across NCI60 cell lines. The tree was inferred using the UPGMA (‘average’) method and was based on Euclidean distances. The tree is presented as an unrooted radial phylogram. The scale in the top left corner is provided for the branch lengths, which were derived from Euclidean distances. Clustered products are displayed with sparse labeling, in which only a random subset of INP labels is displayed. [file 12885_2022_9580_MOESM2_ESM.pdf]
