# Supplementary material for: Pharmacogenomics of in vitro response of the NCI-60 cancer cell line panel to Indian natural products
Source: BMC Cancer. 2022 May 7;22:512. doi: 10.1186/s12885-022-09580-7 (PMC9077913; doi:10.1186/s12885-022-09580-7)

Supp Figure 5

A. Pathways Positively Associated with Subtree 1

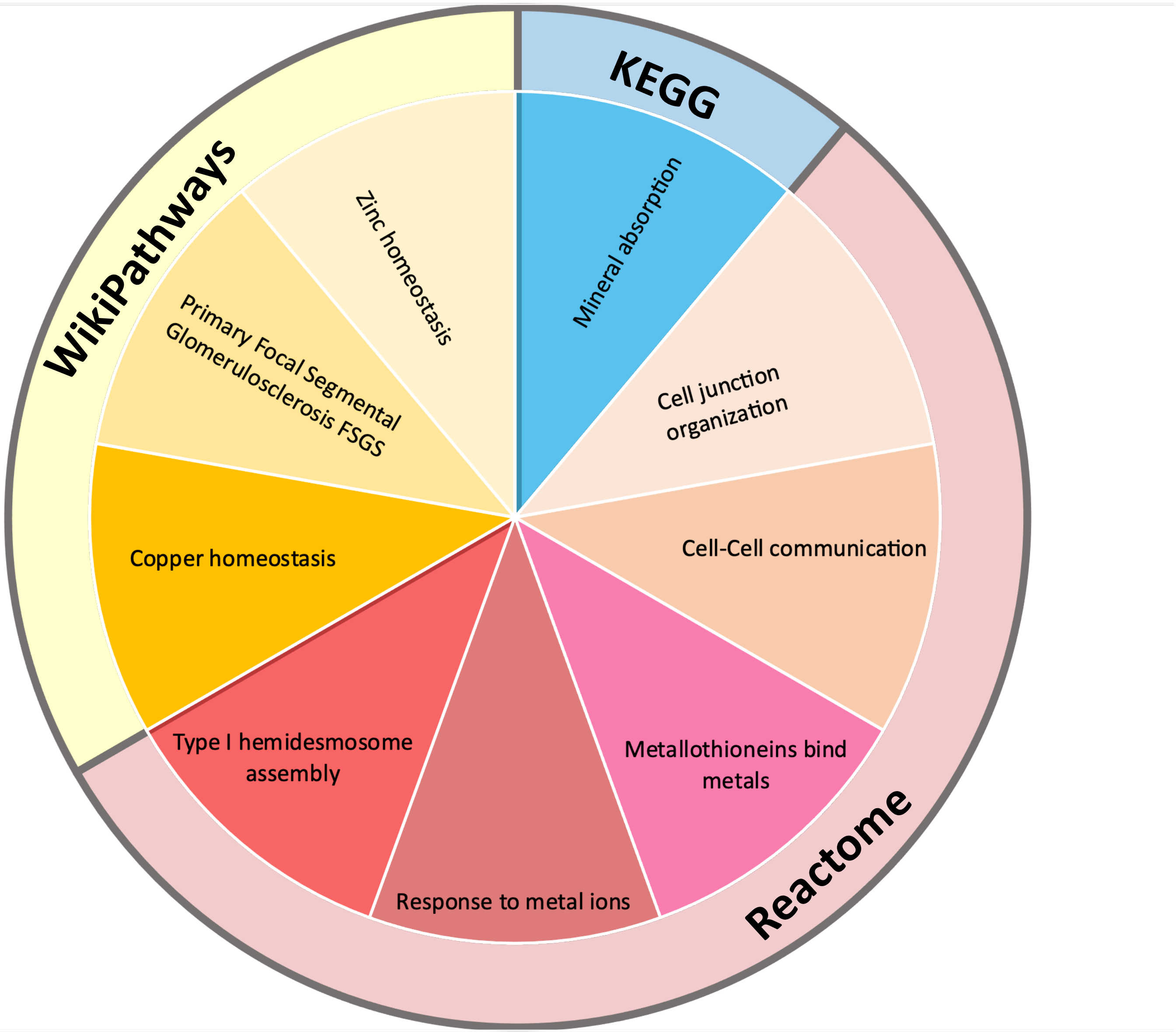

B. Pathways Positively Associated with Subtree 3

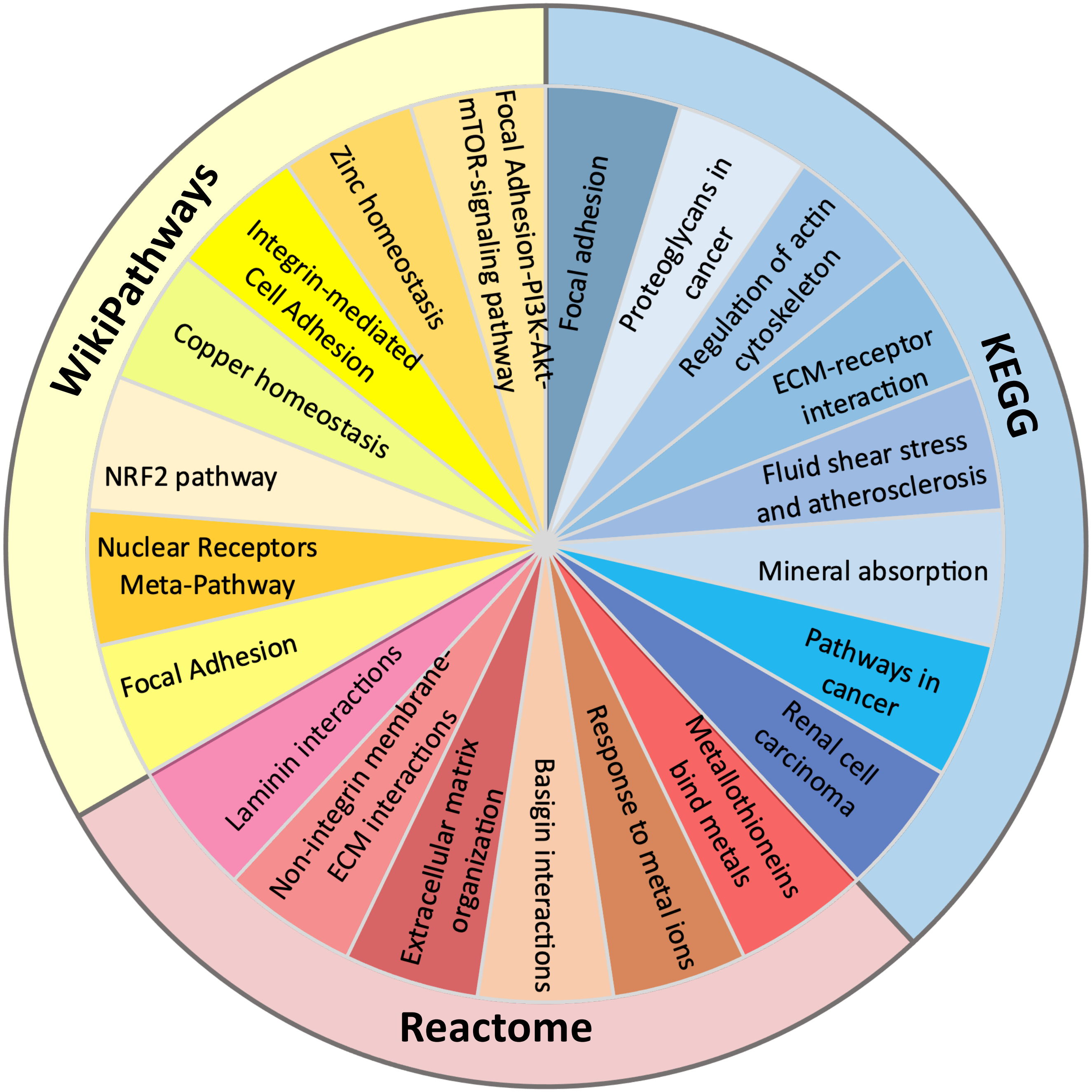

C. Pathways Negatively Associated with Subtree 3

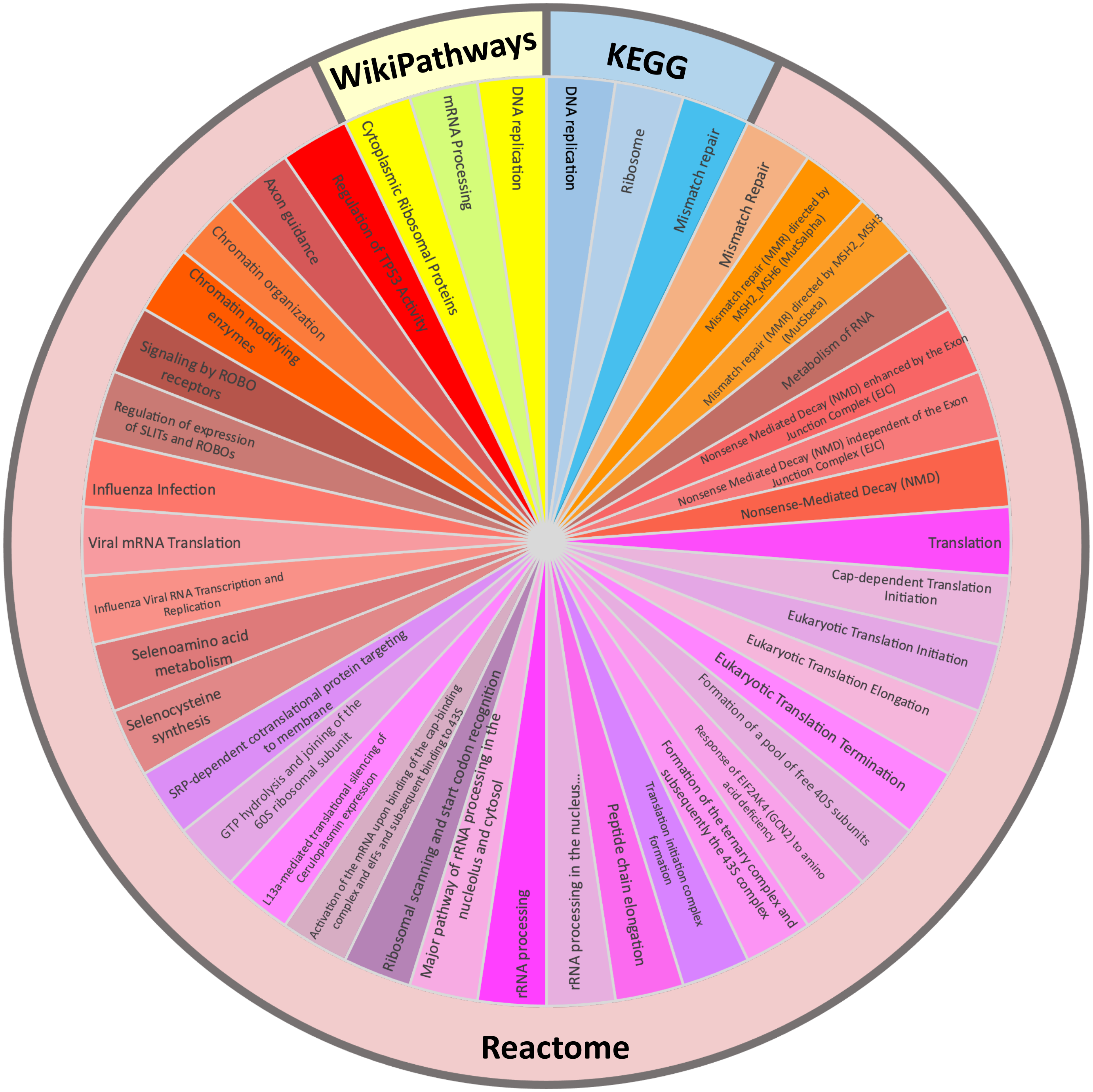

Supplement: Supplementary file 5 — Additional file 5. Supplementary Figure 5. Graphical overview of significant associations of logGI50 of Indian natural product subtrees 1 and 3 with molecular pathways from Reactome, KEGG, and WikiPathways. Shown are significant associations identified by g:Profiler with FDR adjusted p < 0.05. (A) Positive associations for Subtree 1. (B) Positive associations for Subtree 3. (C) Negative associations for Subtree 3. Additional information about each association shown in the Figure is provided in Supplementary Tables 1-3. [file 12885_2022_9580_MOESM5_ESM.pdf]
