# Supplementary material for: Pharmacogenomics of in vitro response of the NCI-60 cancer cell line panel to Indian natural products
Source: BMC Cancer. 2022 May 7;22:512. doi: 10.1186/s12885-022-09580-7 (PMC9077913; doi:10.1186/s12885-022-09580-7)
Supplement: Supplementary file 7 — Additional file 7. Supplementary Table 1: Positively correlated pathways in Subtree 1 [file 12885_2022_9580_MOESM7_ESM.pdf]

Supplementary Table 1: Positively correlated pathways in Subtree 1

Source	Pathway name	Term Id	Adjusted p value	Term Size	Query Size	Intersection Size	Effective Domain Size	Intersections
KEGG	Mineral absorption	KEGG:04978	0.0003247	59	59	6	7963	MT2A,MT1H,MT1HL1,MT1E,MT1B,ATP1A1
REAC	Metallothioneins bind metals	REAC:R-HSA-5661231	0.0000024	11	75	5	10627	MT2A,MT3,MT1H,MT1E,MT1B
REAC	Response to metal ions	REAC:R-HSA-5660526	0.0000103	14	75	5	10627	MT2A,MT3,MT1H,MT1E,MT1B
REAC	Cell junction organization	REAC:R-HSA-446728	0.0011644	91	75	7	10627	LAMC2,PARD3,MPP5,CLDN1,PLEC,ITGB1,CD151
REAC	Cell-Cell communication	REAC:R-HSA-1500931	0.0105032	127	75	7	10627	LAMC2,PARD3,MPP5,CLDN1,PLEC,ITGB1,CD151
REAC	Type I hemidesmosome assembly	REAC:R-HSA-446107	0.0188358	11	75	3	10627	LAMC2,PLEC,CD151
WP	Copper homeostasis	WP:WP3286	0.0007236	54	64	6	7474	MT2A,MT3,MT1H,MT1E,MT1B,ADAM9
WP	Zinc homeostasis	WP:WP3529	0.0017245	37	64	5	7474	MT2A,MT3,MT1H,MT1E,MT1B
WP	Primary Focal Segmental Glomerulosclerosis FSGS	WP:WP2572	0.0499642	74	64	5	7474	ITGA3,AGRN,CLDN1,ITGB1,CD151
