# Supplementary material for: Pharmacogenomics of in vitro response of the NCI-60 cancer cell line panel to Indian natural products
Source: BMC Cancer. 2022 May 7;22:512. doi: 10.1186/s12885-022-09580-7 (PMC9077913; doi:10.1186/s12885-022-09580-7)
Supplement: Supplementary file 8 — Additional file 8. Supplementary Table 2: Positively correlated pathways in Subtree 3 [file 12885_2022_9580_MOESM8_ESM.pdf]

Supplementary Table 2: Positively correlated pathways in Subtree 3

Source	Pathway name	Term Id	Adjusted p value	Term Size	Query Size	Intersection Size	Effective Domain Size	Intersections
KEGG	Focal adhesion	KEGG:04510	0.0000001	200	58	13	7963	LAMB2,THBS1,PDGFC,ACTN4,CAV2,ITGAV,PAK4,CRKL,PDGFD,COL4A1,CAV1,ITGA3,CAPN2
KEGG	Proteoglycans in cancer	KEGG:05205	0.0011162	205	58	9	7963	RDX,THBS1,HIF1A,GPC1,SDC1,TGFB2,CAV2,ITGAV,CAV1
KEGG	Regulation of actin cytoskeleton	KEGG:04810	0.0014605	212	58	9	7963	RDX,PFN4,PDGFC,ACTN4,ITGAV,PAK4,CRKL,PDGFD,ITGA3
KEGG	ECM-receptor interaction	KEGG:04512	0.0029402	88	58	6	7963	LAMB2,THBS1,SDC1,ITGAV,COL4A1,ITGA3
KEGG	Fluid shear stress and atherosclerosis	KEGG:05418	0.0043536	138	58	7	7963	GPC1,SDC1,NQO1,MGST3,CAV2,ITGAV,CAV1

Source	Pathway name	Term Id	Adjusted p value	Term Size	Query Size	Intersection Size	Effective Domain Size	Intersections
KEGG	Mineral absorption	KEGG:04978	0.0047875	59	58	5	7963	ATP1B1,MT1HL1,MT1B,MT1F,MT2A
KEGG	Pathways in cancer	KEGG:05200	0.0062713	529	58	13	7963	TXNRD1,EPAS1,LAMB2,GNA11,HIF1A,GNG11,TGFB2,NQO1,MGST3,ITGAV,CRKL,COL4A1,ITGA3
KEGG	Renal cell carcinoma	KEGG:05211	0.0094829	68	58	5	7963	EPAS1,HIF1A,TGFB2,PAK4,CRKL
REAC	Metallothioneins bind metals	REAC:R-HSA-5661231	0.0002076	11	69	4	10627	MT1B,MT1F,MT2A,MT3
REAC	Response to metal ions	REAC:R-HSA-5660526	0.0006205	14	69	4	10627	MT1B,MT1F,MT2A,MT3

Source	Pathway name	Term Id	Adjusted p value	Term Size	Query Size	Intersection Size	Effective Domain Size	Intersections
REAC	Basigin interactions	REAC:R-HSA-210991	0.0074308	25	69	4	10627	ATP1B1,CAV1,ITGA3,SLC7A11
REAC	Extracellular matrix organization	REAC:R-HSA-1474244	0.0081290	298	69	10	10627	LAMB2,THBS1,CAST,SDC1,TGFB2,ITGAV,CTSD,COL4A1,ITGA3,CAPN2
REAC	Non-integrin membrane-ECM interactions	REAC:R-HSA-3000171	0.0139865	58	69	5	10627	LAMB2,THBS1,SDC1,ITGAV,COL4A1
REAC	Laminin interactions	REAC:R-HSA-3000157	0.0157090	30	69	4	10627	LAMB2,ITGAV,COL4A1,ITGA3
WP	Focal Adhesion	WP:WP306	0.0000019	201	66	13	7474	LAMB2,THBS1,PDGFC,ACTN4,CAV2,ITGAV,PAK4,CRKL,PDGFD,COL4A1,CAV1,ITGA3,CAPN2

Source	Pathway name	Term Id	Adjusted p value	Term Size	Query Size	Intersection Size	Effective Domain Size	Intersections
WP	Nuclear Receptors Meta-Pathway	WP:WP2882	0.0023862	321	66	12	7474	TXNRD1,PTGR1,EPB41L4B,MYOF,TGFB2,NQO1,LRRC8A,MGST3,SRXN1,G6PD,ETNK2,SLC7A11
WP	NRF2 pathway	WP:WP2884	0.0044892	145	66	8	7474	TXNRD1,PTGR1,TGFB2,NQO1,MGST3,SRXN1,G6PD,SLC7A11
WP	Copper homeostasis	WP:WP3286	0.0127479	54	66	5	7474	MT1B,MT1F,MT2A,MT3,MT1JP
WP	Integrin-mediated Cell Adhesion	WP:WP185	0.0336649	103	66	6	7474	CAV2,ITGAV,PAK4,CAV1,ITGA3,CAPN2
WP	Zinc homeostasis	WP:WP3529	0.0359739	37	66	4	7474	MT1B,MT1F,MT2A,MT3

Source	Pathway name	Term Id	Adjusted p value	Term Size	Query Size	Intersection Size	Effective Domain Size	Intersections
WP	Focal Adhesion-PI3K-Akt-mTOR-signaling pathway	WP:WP3932	0.0404159	308	66	10	7474	EPAS1,LAMB2,THBS1,PDGFC,HIF1A,GNG11,ITGAV,PDGFD,COL4A1,ITGA3
