# Supplementary material for: Pharmacogenomics of in vitro response of the NCI-60 cancer cell line panel to Indian natural products
Source: BMC Cancer. 2022 May 7;22:512. doi: 10.1186/s12885-022-09580-7 (PMC9077913; doi:10.1186/s12885-022-09580-7)
Supplement: Supplementary file 9 — Additional file 9. Supplementary Table 3: Negatively correlated pathways in Subtree 3 [file 12885_2022_9580_MOESM9_ESM.pdf]

Supplementary Table 3: Negatively correlated pathways in Subtree 3

Source	Pathway name	Term Id	Adjusted p value	Term Size	Query Size	Intersection Size	Effective Domain Size	Intersections
KEGG	Ribosome	KEGG:03010	0.0000001	153	60	12	7963	RPL11,RPSA,RPL34,RPS10,RPL10A,RSL24D1,MRPL16,RPL39,RPS27A,RPL6,RPS9,RPS25
KEGG	Mismatch repair	KEGG:03430	0.0499254	23	60	3	7963	POLD2,POLD1,MSH2
KEGG	DNA replication	KEGG:03030	0.1877628	36	60	3	7963	POLD2,POLD1,MCM7
REAC	Metabolism of RNA	REAC:R-HSA-8953854	0.0000000	661	74	24	10627	PDCD11,ADAT2,HNRNPR,RPL11,RPSA,PNRC2,RPL34,RPS10,HNRNPM,RPL10A,SRRM1,RPL39,DDX21,RPS27A,RPL6,SRSF10,SRRT,SNRNP40,NUP88,RPS9,TRMU,NAT10,LSM2,RPS25
REAC	Nonsense Mediated Decay (NMD) enhanced by the Exon Junction Complex (EJC)	REAC:R-HSA-975957	0.0000001	116	74	11	10627	RPL11,RPSA,PNRC2,RPL34,RPS10,RPL10A,RPL39,RPS27A,RPL6,RPS9,RPS25
REAC	Nonsense-Mediated Decay (NMD)	REAC:R-HSA-927802	0.0000001	116	74	11	10627	RPL11,RPSA,PNRC2,RPL34,RPS10,RPL10A,RPL39,RPS27A,RPL6,RPS9,RPS25
REAC	Viral mRNA Translation	REAC:R-HSA-192823	0.0000002	90	74	10	10627	RPL11,RPSA,RPL34,RPS10,RPL10A,RPL39,RPS27A,RPL6,RPS9,RPS25

Source	Pathway name	Term Id	Adjusted p value	Term Size	Query Size	Intersection Size	Effective Domain Size	Intersections
REAC	Peptide chain elongation	REAC:R-HSA-156902	0.0000002	90	74	10	10627	RPL11,RPSA,RPL34,RPS10,RPL10A,RPL39,RPS27A,RPL6,RPS9,RPS25
REAC	rRNA processing in the nucleus and cytosol	REAC:R-HSA-8868773	0.0000002	193	74	13	10627	PDCD11,RPL11,RPSA,RPL34,RPS10,RPL10A,RPL39,DDX21,RPS27A,RPL6,RPS9,NAT10,RPS25
REAC	Selenocysteine synthesis	REAC:R-HSA-2408557	0.0000003	94	74	10	10627	RPL11,RPSA,RPL34,RPS10,RPL10A,RPL39,RPS27A,RPL6,RPS9,RPS25
REAC	Eukaryotic Translation Elongation	REAC:R-HSA-156842	0.0000003	94	74	10	10627	RPL11,RPSA,RPL34,RPS10,RPL10A,RPL39,RPS27A,RPL6,RPS9,RPS25
REAC	Eukaryotic Translation Termination	REAC:R-HSA-72764	0.0000003	94	74	10	10627	RPL11,RPSA,RPL34,RPS10,RPL10A,RPL39,RPS27A,RPL6,RPS9,RPS25
REAC	Nonsense Mediated Decay (NMD) independent of the Exon Junction Complex (EJC)	REAC:R-HSA-975956	0.0000004	96	74	10	10627	RPL11,RPSA,RPL34,RPS10,RPL10A,RPL39,RPS27A,RPL6,RPS9,RPS25
REAC	rRNA processing	REAC:R-HSA-72312	0.0000004	203	74	13	10627	PDCD11,RPL11,RPSA,RPL34,RPS10,RPL10A,RPL39,DDX21,RPS27A,RPL6,RPS9,NAT10,RPS25

Source	Pathway name	Term Id	Adjusted p value	Term Size	Query Size	Intersection Size	Effective Domain Size	Intersections
REAC	Influenza Viral RNA Transcription and Replication	REAC:R-HSA-168273	0.0000006	132	74	11	10627	RPL11,RPSA,RPL34,RPS10,RPL10A,RPL39,RPS27A,RPL6,NUP88,RPS9,RPS25
REAC	Formation of a pool of free 40S subunits	REAC:R-HSA-72689	0.0000007	102	74	10	10627	RPL11,RPSA,RPL34,RPS10,RPL10A,RPL39,RPS27A,RPL6,RPS9,RPS25
REAC	Response of EIF2AK4 (GCN2) to amino acid deficiency	REAC:R-HSA-9633012	0.0000007	102	74	10	10627	RPL11,RPSA,RPL34,RPS10,RPL10A,RPL39,RPS27A,RPL6,RPS9,RPS25
REAC	Major pathway of rRNA processing in the nucleolus and cytosol	REAC:R-HSA-6791226	0.0000015	183	74	12	10627	PDCD11,RPL11,RPSA,RPL34,RPS10,RPL10A,RPL39,DDX21,RPS27A,RPL6,RPS9,RPS25
REAC	L13a-mediated translational silencing of Ceruloplasmin expression	REAC:R-HSA-156827	0.0000016	112	74	10	10627	RPL11,RPSA,RPL34,RPS10,RPL10A,RPL39,RPS27A,RPL6,RPS9,RPS25

Source	Pathway name	Term Id	Adjusted p value	Term Size	Query Size	Intersection Size	Effective Domain Size	Intersections
REAC	GTP hydrolysis and joining of the 60S ribosomal subunit	REAC:R-HSA-72706	0.0000018	113	74	10	10627	RPL11,RPSA,RPL34,RPS10,RPL10A,RPL39,RPS27A,RPL6,RPS9,RPS25
REAC	SRP-dependent cotranslational protein targeting to membrane	REAC:R-HSA-1799339	0.0000018	113	74	10	10627	RPL11,RPSA,RPL34,RPS10,RPL10A,RPL39,RPS27A,RPL6,RPS9,RPS25
REAC	Influenza Infection	REAC:R-HSA-168255	0.0000023	151	74	11	10627	RPL11,RPSA,RPL34,RPS10,RPL10A,RPL39,RPS27A,RPL6,NUP88,RPS9,RPS25
REAC	Selenoamino acid metabolism	REAC:R-HSA-2408522	0.0000025	117	74	10	10627	RPL11,RPSA,RPL34,RPS10,RPL10A,RPL39,RPS27A,RPL6,RPS9,RPS25
REAC	Cap-dependent Translation Initiation	REAC:R-HSA-72737	0.0000032	120	74	10	10627	RPL11,RPSA,RPL34,RPS10,RPL10A,RPL39,RPS27A,RPL6,RPS9,RPS25
REAC	Eukaryotic Translation Initiation	REAC:R-HSA-72613	0.0000032	120	74	10	10627	RPL11,RPSA,RPL34,RPS10,RPL10A,RPL39,RPS27A,RPL6,RPS9,RPS25

Source	Pathway name	Term Id	Adjusted p value	Term Size	Query Size	Intersection Size	Effective Domain Size	Intersections
REAC	Regulation of expression of SLITs and ROBOs	REAC:R-HSA-9010553	0.0000951	171	74	10	10627	RPL11,RPSA,RPL34,RPS10,RPL10A,RPL39,RPS27A,RPL6,RPS9,RPS25
REAC	Translation	REAC:R-HSA-72766	0.0002546	292	74	12	10627	RPL11,RPSA,RPL34,RPS10,RPL10A,MRPL16,RPL39,RPS27A,RPL6,RPS9,MARS2,RPS25
REAC	Signaling by ROBO receptors	REAC:R-HSA-376176	0.0008459	217	74	10	10627	RPL11,RPSA,RPL34,RPS10,RPL10A,RPL39,RPS27A,RPL6,RPS9,RPS25
REAC	Chromatin modifying enzymes	REAC:R-HSA-3247509	0.0009410	273	74	11	10627	SMARCC1,HIST1H2AH,HDAC10,ELP2,MSL2,HDAC1,KDM1A,EHMT2,KDM2B,CHD4,YEATS4
REAC	Chromatin organization	REAC:R-HSA-4839726	0.0009410	273	74	11	10627	SMARCC1,HIST1H2AH,HDAC10,ELP2,MSL2,HDAC1,KDM1A,EHMT2,KDM2B,CHD4,YEATS4
REAC	Formation of the ternary complex, and subsequently, the 43S complex	REAC:R-HSA-72695	0.0103457	52	74	5	10627	RPSA,RPS10,RPS27A,RPS9,RPS25

Source	Pathway name	Term Id	Adjusted p value	Term Size	Query Size	Intersection Size	Effective Domain Size	Intersections
REAC	Translation initiation complex formation	REAC:R-HSA-72649	0.0191888	59	74	5	10627	RPSA,RPS10,RPS27A,RPS9,RPS25
REAC	Ribosomal scanning and start codon recognition	REAC:R-HSA-72702	0.0191888	59	74	5	10627	RPSA,RPS10,RPS27A,RPS9,RPS25
REAC	Activation of the mRNA upon binding of the cap-binding complex and eIFs, and subsequent binding to 43S	REAC:R-HSA-72662	0.0208204	60	74	5	10627	RPSA,RPS10,RPS27A,RPS9,RPS25
REAC	Axon guidance	REAC:R-HSA-422475	0.0333627	549	74	13	10627	RPL11,RPSA,RPL34,RPS10,RPL10A,RPL39,VLDLR,RPS27A,RPL6,TLN1,RPS9,SIAH2,RPS25
REAC	Mismatch repair (MMR) directed by MSH2:MSH6 (MutSalpha)	REAC:R-HSA-5358565	0.0401662	14	74	3	10627	POLD2,POLD1,MSH2

Source	Pathway name	Term Id	Adjusted p value	Term Size	Query Size	Intersection Size	Effective Domain Size	Intersections
REAC	Mismatch repair (MMR) directed by MSH2:MSH3 (MutSbeta)	REAC:R-HSA-5358606	0.0401662	14	74	3	10627	POLD2,POLD1,MSH2
REAC	Regulation of TP53 Activity	REAC:R-HSA-5633007	0.0427198	160	74	7	10627	NOC2L,TAF4B,RPS27A,TAF5,HDAC1,EHMT2,CHD4
REAC	Mismatch Repair	REAC:R-HSA-5358508	0.0499568	15	74	3	10627	POLD2,POLD1,MSH2
WP	Cytoplasmic Ribosomal Proteins	WP:WP477	0.0000002	90	59	10	7474	RPL11,RPSA,RPL34,RPS10,RPL10A,RPL39,RPS27A,RPL6,RPS9,RPS25
WP	mRNA Processing	WP:WP411	0.0092882	133	59	7	7474	SRPK1,HNRNPR,HNRNPM,SRRM1,SRSF10,SNRNP40,LSM2
WP	DNA Replication	WP:WP466	0.0382271	42	59	4	7474	POLD2,POLD1,MCM7,ORC1
