# Supplementary material for: Pharmacogenomics of in vitro response of the NCI-60 cancer cell line panel to Indian natural products
Source: BMC Cancer. 2022 May 7;22:512. doi: 10.1186/s12885-022-09580-7 (PMC9077913; doi:10.1186/s12885-022-09580-7)
Supplement: Supplementary file 10 — Additional file 10. Supplementary Table 4: All queried Ayurvedic INPs from the PUBLIC COMPARE portal [file 12885_2022_9580_MOESM10_ESM.pdf]

Supplementary Table 4: All queried Ayurvedic INPs from the PUBLIC COMPARE portal

INP name	Ayurvedic Herb/Species Name	NSC number
Curcumin-difluorinated (CDF)	Turmeric/Curcuma longa	752571
Di-O-Adamantoylcurcumin	Turmeric/Curcuma longa	742024
Di-O-(2-Thienoyl)curcumin	Turmeric/Curcuma longa	742023
Curcumin tri trithiadiazolaminoethylcarbonate	Turmeric/Curcuma longa	742022
Curcumin tri adamantylaminoethylcarbonate	Turmeric/Curcuma longa	742021
Ethoxycurcumin trithiadiazolaminomethylcarbonte	Turmeric/Curcuma longa	742020
Ethoxycurcumin tribenzimidazolmethylcarbonte	Turmeric/Curcuma longa	742019
Cucurbitacin-L-2-O-.beta.-D-glucopyranoside	Gourd/Cucurbitaceae	710355
Curcumenol	Turmeric/Curcuma longa	682343
Cucurbitacin E 2-O-glucoside	Gourd/Cucurbitaceae	680118
B820585K273-F221 11-Deoxocucurbitacin I	Gourd/Cucurbitaceae	605109
CUCURBITACIN I	Gourd/Cucurbitaceae	521777
CUCURBITACIN D	Gourd/Cucurbitaceae	521776
CUCURBITACIN E	Gourd/Cucurbitaceae	521775
CUCURBITACIN B, 3 -EPI-ISO-	Gourd/Cucurbitaceae	359240
16-Epicucurbitacin B	Gourd/Cucurbitaceae	350002
CUCURBITACIN GLYCOSIDE DERIV B820585K205	Gourd/Cucurbitaceae	337579
CUCURBITACIN GLYCOSIDE DERIV B820585K203	Gourd/Cucurbitaceae	337578
CUCURBITACIN D	Gourd/Cucurbitaceae	308606
CUCURBITACIN Q-1	Gourd/Cucurbitaceae	306687
ISOCUCURBITACIN D, 3-EPI-	Gourd/Cucurbitaceae	305982
ISOCUCURBITACIN D	Gourd/Cucurbitaceae	305981
Cucurbitacin I, 2-glucoside	Gourd/Cucurbitaceae	184734
Cucurbitacin B	Gourd/Cucurbitaceae	144154
CUCURBITACIN D DEHYDROEPIRHAMNOSIDE	Gourd/Cucurbitaceae	144153
Cucurbitacin Q	Gourd/Cucurbitaceae	135075
CUCURBITACIN P	Gourd/Cucurbitaceae	135074
Cucurbitacine (i)	Gourd/Cucurbitaceae	112167
Cucurbitacin K	Gourd/Cucurbitaceae	112166
Cucurbitacin J	Gourd/Cucurbitaceae	112165
Tetrahydrocucurbitacin I	Gourd/Cucurbitaceae	112164
Cucurbitacin B, dihydro-	Gourd/Cucurbitaceae	106401
Isocucurbitacin B	Gourd/Cucurbitaceae	106400
Cucurbitacine E	Gourd/Cucurbitaceae	106399
Cucurbitacin C	Gourd/Cucurbitaceae	94744

Cucurbitacin A	Gourd/Cucurbitaceae	94743
CUCURBITACIN B	Gourd/Cucurbitaceae	49451
Curcuma	Turmeric/Curcuma longa	32982
Curcumoid	Turmeric/Curcuma longa	26727
Oroxylon indicum (alc. extract - leaf,stem,fruit)	Shyonaka- Root bark/Oroxylum indicum	20362
Rauwolfia tetraphylla (hot alc. extract - leaf,flowers)	Sarpagandha/Rauvolfia serpentina	14897
7,12-Dimethylbenz[a]anthracene	Ashoka tree/Saraca indica	408823
Heterophylline (Rauwolfia)	Sarpagandha/Rauvolfia serpentina	72136
Rauwolfin	Sarpagandha/Rauvolfia serpentina	15627
Rauwolfia serpentina (alkaloidal, extract of)	Sarpagandha/Rauvolfia serpentina	15625
Elagic Acid	Pomegranate flower/ Punicoideae	656272
Piperlonguminine	Pippali/Piperaceae	125178
Hypophyllanthin	Phyllanthus	619044
Phyllanthin	Phyllanthus	619043
Phyllanthostatin A B680433K421	Phyllanthus	618898
Beta.-D-Lactosyl-1-amidophyllanthocin	Phyllanthus	615436
Beta.-D-Cellobiosyl-1-amidophyllanthocin	Phyllanthus	615435
PHYLLANTHOSIDE, S4,S4'-DIACETATE	Phyllanthus	349989
PHYLLANTHOSIDE S3-DESACETYL	Phyllanthus	349988
PHYLLANTHOSIDE, S3,S4'-DIACETATE	Phyllanthus	349987
PHYLLANTHOSTATIN 3 AGLYCONE METHYLESTER	Phyllanthus	349984
PHYLLANTHOSE (DISACCHARIDE FROM PHYLLANTHOSIDE)	Phyllanthus	348102
PHYLLANTHOSTATIN 1,S3'-DESACETYL	Phyllanthus	348101
PHYLLANTHOSIDE PERACETATE	Phyllanthus	345396
PHYLLANTHOCIN	Phyllanthus	345395
PHYLLANTHOSIDE, DIDESACETYL	Phyllanthus	342734
PHYLLANTHOSIDE, S3'-DESACETYL-	Phyllanthus	342443
Phyllanthastatin 1	Phyllanthus	332596
Phyllanthostatin 3	Phyllanthus	332189
From plant Phyllanthus brasiliensis	Phyllanthus	330498
Phyllanthastatin 2	Phyllanthus	329098
PHYLLANTHOSIDE	Phyllanthus	328426
PHYLLANTHOSIDE, CRUDE	Phyllanthus	318507
Phyllanthoside	Phyllanthus	266492
Saffron	Oils, saffron/Crocus Sativus	407300
AZADIRACHTIN	Neem/ Azadirachta indica	368675
Leurosine	Madagascar periwinkle/Catharanthus roseus	90636
SYMPLOCOSIN, HEXAHYDRATE	Lodhra/Symplocos Racemosa	35608

Extract from Glycyrrhiza shiheziensis plant	Licorice/ Glycyrrhiza	637466
Extract from Glycyrrhiza alaernsis plant	Licorice/ Glycyrrhiza	637465
Extract from Glycyrrhiza inflata plant	Licorice/ Glycyrrhiza	637464
Extract from Glycyrrhiza macrophylla plant	Licorice/ Glycyrrhiza	637463
Glycyrrhizin	Licorice/ Glycyrrhiza	234419
Glycyrrhizin	Licorice/ Glycyrrhiza	167409
Glycyrrhizic acid monosodium salt	Licorice/ Glycyrrhiza	163964
Glycyrrhizinic acid	Licorice/ Glycyrrhiza	35348
Glycyrrhizinic acid, ammonium salt (1:1)	Licorice/ Glycyrrhiza	2800
Gloriosine	Kalapikilangu/Gloriosa superba	403142
Buddhist Bauhinia	Kachnar/Bauhinia	20436
Marmelosin	Bael fruit/Aegle marmelos	402949
Symplocos paniculata (alc. extract - leaf)	Sapphire Berry/Symplocos paniculata	19238
Terminalia chebula myrobalans ext.	Haritaki/ Terminalia chebula	11099
Commiphora, polysaccharide from	Guggal/ Commiphora	407678
Unbleached ginger - Zingiber officinale (Zingiberaceae)	Ginger/Zingiberaceae	745202
Zingiberone	Ginger/Zingiberaceae	15335
Embelin	False black pepper/Embelia Ribus	91874
Isobromodeoxytopsentin	Elephant’s foot/Elephantopus scaber	713185
Daturaolone	Angel’s Trumpet/Datura	705537
DATURALACTONE	Angel’s Trumpet/Datura	285115
Citral	Lemongrass/Cymbopogon citrates	6170
CUCURBITACIN F	Gourd/Cucurbitaceae	251680
Lupeol	Three leaves caper/Crataeva nurvala	90487
Fagarasterol (stem bark)	Three leaves caper/Crataeva nurvala	90487
Diethyl p-trifluoromethyl-.alpha.-phosphonocinnamonitrile	Cinnamon/Cinnamonum	648749
Diethyl m-phenoxy-.alpha.-phosphonocinnamonitrile	Cinnamon/Cinnamonum	648748
p-Phenoxy-diethylphosphonocinnamonitrile	Cinnamon/Cinnamonum	648747
o-Fluoro-.alpha.-benzoyl cinnamonitrile	Cinnamon/Cinnamonum	643772
o-Bromo-.alpha.-benzoyl cinnamonitrile	Cinnamon/Cinnamonum	643769
o-Methoxy-.alpha.-benzoylcinnamonitrile	Cinnamon/Cinnamonum	643764
3-Methoxy-4-benzyloxy-.alpha.-benzoylcinnamonitrile	Cinnamon/Cinnamonum	643190
3,5-Dimethoxy-.alpha.-phenylcinnamonitrile	Cinnamon/Cinnamonum	643185
3-Methoxy-4-hydroxy-.alpha.-benzoylcinnamonitrile	Cinnamon/Cinnamonum	643183
3,4,5-Trimethoxy-.alpha.-benzoyl cinnamonitrile	Cinnamon/Cinnamonum	643181
Diethyl 3,4-methylenedioxy-.alpha.-phosphono cinnamonitrile	Cinnamon/Cinnamonum	643169

3,4-Methylenedioxy-.alpha.-benzoyl cinnamonitrile	Cinnamon/Cinnamonum	643167
3-Bromo-4-dimethylamino-.alpha.-benzoyl cinnamonitrite	Cinnamon/Cinnamonum	643160
p-Acetoxy-.alpha.-diethylphosphono-cinnamonitrile	Cinnamon/Cinnamonum	643033
m-Phenoxy-.alpha.-phenylcinnamonitrile	Cinnamon/Cinnamonum	643032
Alpha.-Phenyl-2,5-dimethoxy-.alpha.-cinnamonitrile	Cinnamon/Cinnamonum	643023
Hydrocinnamonitrile, .alpha.,3-dichloro-2-methyl-|8th C.I.	Cinnamon/Cinnamonum	239071
Cinnamonitrile, p-(dimethylamino)-|8th C.I.	Cinnamon/Cinnamonum	122722
3-Phenylcinnamonitrile	Cinnamon/Cinnamonum	120378
Hydrocinnamonitrile, p-cyano-|8th C.I.	Cinnamon/Cinnamonum	117833
(E)-Cinnamonitrile	Cinnamon/Cinnamonum	77496
Cinnamonitrile, 3,4-dimethoxy-|8th C.I.	Cinnamon/Cinnamonum	51968
CINNAMONITRILE	Cinnamon/Cinnamonum	49137
Alpha.,.beta.-Diphenylcinnamonitrile	Cinnamon/Cinnamonum	42900
Cinnamonitrile	Cinnamon/Cinnamonum	42118
Cinnamonfern	Cinnamon/Cinnamonum	17993
Hydrocinnamonitrile	Cinnamon/Cinnamonum	16936
Alpha.-Phenylcinnamonitrile	Cinnamon/Cinnamonum	12489
Acetocinnamone	Cinnamon/Cinnamonum	5605
Hydrocinnamonitrile, .beta.-oxo-	Cinnamon/Cinnamonum	4713
Alpha.-Phenylcinnamonitrile	Cinnamon/Cinnamonum	2018
Alpha.-Cyanocinnamonitrile	Cinnamon/Cinnamonum	490
Cichoriin aglucon	Cichory/ Asteraceae	26428
Statice limonium (Plumbaginaceae), water extract	Chitraka/Plumbago zeylanica	179851
Statice sinuata (Plumbaginaceae), methanol extract	Chitraka/Plumbago zeylanica	179850
Plumbagin	Chitraka/Plumbago zeylanica	688284
Plumbagin, 3-bromo:	Chitraka/Plumbago zeylanica	621605
CHLOROPLUMBAGIN	Chitraka/Plumbago zeylanica	362426
PLUMBAGIN	Chitraka/Plumbago zeylanica	236613
Calotropin	Arka/Calotropis Gigantea	143925
Calendulaglycoside D-6'-O-methyl ester	Marigold/Calendula	731922
Calendulaglycoside D2	Marigold/Calendula	731921
Calendulaglycoside B-6'-O-butyl ester	Marigold/Calendula	731920
Calendulaglycoside A-6'-O-butyl ester	Marigold/Calendula	731919
Calendulaglycoside C	Marigold/Calendula	731918
Calendulaglycoside B	Marigold/Calendula	731917
Calendulaglycoside A	Marigold/Calendula	731916
Faradione, from Calendula	Marigold/Calendula	277277
Calendula acids	Marigold/Calendula	119126
Butein	Palasha/Butea monosperma	652892

Isolated from Asteraceae plants	Asteraceae	603929
Achilleol A	Biranjasipha/ Achillea millefolium	710351
DEHYDROACHILLIN	Biranjasipha/ Achillea millefolium	180034
Achillin	Biranjasipha/ Achillea millefolium	156236
Achillea millefolium (chloroform extract)	Biranjasipha/ Achillea millefolium	18444
Achillea millefolium plant chloroform extract	Biranjasipha/ Achillea millefolium	17263
Guamachil Apes-earring	Biranjasipha/ Achillea millefolium	14900
Achilleic acid	Biranjasipha/ Achillea millefolium	7616
Phyllanthus emblica (alc. extract - leaf,fruit)	Bhumi amalaka/Phyllanthus	20426
Terminalia bellenca (hot alcohol extract- leaf, stem, fruit) Terminalia	Bhibhitaki/ Terminalia bellenca	20331
Nimbolide	Neem/Azadirachta indica	309909
WITHANIA SOMNIFERA	Aswagandha/Withania Somnifera	329512
Common Saraca	Asoka/Saraca Asoca	20482
Terminalia chebula (alc. extract - leaf, inflor.)	Arjuna/ Arjuna Terminalia	18003
Ricinusol	Argan Fruits/Argania spinosa	360316
Ricinus communis	Argan Fruits/Argania spinosa	15384
Andrographis Extract	Kalmegh/Andrographis Paniculata	383468
Myricetin	Neem/Azadirachta indica	407290
Barbaloin	Aloe vera (Aloe)/Asphodeloideae	227189
Vasicine	Adhatoda vasica	28595
Perchlorate of an alkaloid from Rutaceae	Aegle marmelos	342730
Alkaloid from Rutaceae found in Cameroon	Aegle marmelos	342729
Bulbophyllanthrone	Bulbophyllum odoratissimum/ORCHIDACEAE	708791
Rubia tenuifolia water extract	Indian madder/ Rubia tenuifolia	179855
Rubia tenuifolia chloroform ext.	Indian madder/ Rubia tenuifolia	179854
Tylophorine	Tylophora asthmatica	717335
Tylophorine	Tylophora asthmatica	76387
Cryptoleurine	Tylophora	19912
Limonene	Citrus peels/Anethum graveolens	21446
Limonene	Citrus peels/Anethum graveolens	844
Caffeic acid	Honey, coffee	57197
Gallocatechin (Bark Extract)	Arjuna/ Arjuna Terminalia	674038
Gedunin	Neem/Azadirachta indica	113497
Coumestrol	Red clover/Trifolium pratense	22842
Alizarin	Indian madder/Rubia cordifolia	7212
Quercetin	Phyllanthus	9219
Catechin	Green tea	2819
Reservatol	Darakchasava/Vinis vinifera	327430
Piceatannol	Darakchasava/Vinis vinifera	365798

All-trans beta carotene	Mahua/Madhuca indica	62794
Phenethyl Isothiocyanate	Radish/Raphanas sativus	87868
Beta-Sitosterol	Vajradanti/BARLERIA PRIONITIS	8096
Paeoniflorin	Elephant foot yam/ Amorphophallus paeoniifolius	178886

The column **Ayurvedic Herb/Species Name** shows the taxonomic origin for the sources of Ayurvedic compounds. For those compounds that originate from multiple source organisms, only selected examples of source organisms are listed.
